# The Quality Assessment of Different Geolocalisation Methods for a Sensor System to Monitor Structural Health of Monumental Objects

**DOI:** 10.3390/s20102915

**Published:** 2020-05-21

**Authors:** Jakub Markiewicz, Sławomir Łapiński, Patryk Kot, Aleksandra Tobiasz, Magomed Muradov, Joanna Nikel, Andy Shaw, Ahmed Al-Shamma’a

**Affiliations:** 1 Faculty of Geodesy and Cartography, Warsaw University of Technology, Pl. Politechniki 1, 00-661 Warsaw, Poland; Slawomir.Lapinski@pw.edu.pl; 2 Built Environment and Sustainable Technologies (BEST) Research Institute, Faculty of Engineering and Technology, Liverpool John Moores University, Liverpool L3 3AF, UK; P.Kot@ljmu.ac.uk (P.K.); M.Muradov@ljmu.ac.uk (M.M.); A.Shaw@ljmu.ac.uk (A.S.); 3 Documentation and Digitalization Department, Museum of King Jan III’s Palace at Wilanów, ul. Stanisława Kostki Potockiego 10/16, 02-958 Warsaw, Poland; atobiasz@muzeum-wilanow.pl; 4 Department of Material Culture History, University of Wrocław, Szewska 49, 50-137 Wroclaw, Poland; joanna.nikel@uwr.edu.pl; 5 Collage of Engineering, University of Sharjah, Sharjah P.O. Box 27272, UAE; alshammaa@sharjah.ac.ae

**Keywords:** cultural heritage, electromagnetic sensor, geolocalisation, multisensor platform, non-destructive techniques, photogrammetry, Structure-from-Motion, structural health monitoring (SHM), surveying

## Abstract

Cultural heritage objects are affected by a wide range of factors causing their deterioration and decay over time such as ground deformations, changes in hydrographic conditions, vibrations or excess of moisture, which can cause scratches and cracks formation in the case of historic buildings. The electromagnetic spectroscopy has been widely used for non-destructive structural health monitoring of concrete structures. However, the limitation of this technology is a lack of geolocalisation in the space for multispectral architectural documentation. The aim of this study is to examine different geolocalisation methods in order to determine the position of the sensor system, which will then allow to georeference the results of measurements performed by this device and apply corrections to the sensor response, which is a crucial element required for further data processing related to the object structure and its features. The classical surveying, terrestrial laser scanning (TLS), and Structure-from-Motion (SfM) photogrammetry methods were used in this investigation at three test sites. The methods were reviewed and investigated. The results indicated that TLS technique should be applied for simple structures and plain textures, while the SfM technique should be used for marble-based and other translucent or semi-translucent structures in order to achieve the highest accuracy for geolocalisation of the proposed sensor system.

## 1. Introduction

Cultural heritage objects are still endangered by a wide range of factors causing their deterioration and decay over time. In general, degradation processes are caused by both human activities (e.g., pollution, wrong conservation methods, vandalism) and environmental factors [1,2], including the influence of atmospheric conditions and climate change or biological invasion. For this reason, conservatory works are necessary to keep cultural heritage objects and sites in good shape and conditions in order to preserve them for future generations.

Architectural heritage, i.e., antique buildings and small architecture are affected by many degradation processes of varied course, dependent on characteristics of used materials as well as the object’s age, working conditions and random events [3]. The properties of the objects’ material may change over time due to natural processes, which can be influenced and accelerated by the presence of water, temperature variations, and micro-climatic conditions, e.g., pollution [4].

In order to find proper methods of conservation and maintenance of an antique building as well as to establish work schedule, it is necessary to identify causes of the objects’ degradation [5,6]. Tests performed in situ involve diagnosis based on qualitative approach (direct observation of structural damage, historical and archaeological research) and quantitative approach (monitoring and structural analysis) [6]. Diagnostic methods consider damage inspection, material testing and analyses as well as the detection of hidden spaces and cavities [7]. For the identification of wall composition, georadar, and endoscopic tests can be applied, especially in case of detection of voids, cavities, and consolidation interventions [4,7,8]. The presence of moisture in porous material, especially in water-damaged and humid buildings, is one of the factors causing biological invasion: mildew, moulds, and efflorescence. The examination of such forms of decay can be combined with the monitoring of environmental parameters in order to retrace deterioration patterns.

Structural health monitoring (SHM) for historical and architectural heritage should concern such objectives as [9]:

- measuring ambient vibrations induced by traffic, wind etc.;
- measuring the evolution of existing cracks;
- observing the damage coming from chemical, physical and biological degradation of the object’s material.

Electromagnetic spectroscopy offers non-destructive [10] or embedded solutions [11,12] for structural health monitoring. The electromagnetic sensor, which is the focus of this work, has been used for monitoring of the excess moisture content and waterproof membrane defects [13,14] of concrete structures. The sensor demonstrated a good potential as a method for SHM. However, the limitation of this technology is the lack of geolocalisation in space which prevents the resulting data from being used as multispectral architectural documentation [15]. The accuracy of measurements performed by the proposed electromagnetic (EM) sensor relies on accurate positioning of the system owing to the air gap between the sensor and the scanned object as well as the angle of the sensor in relation to the object. These parameters are essential in analysis of obtained data as they influence/alter the reflected signal, i.e., the sensor response. Therefore, the aim of this article is to investigate the most suitable geolocalisation method for the proposed sensor system as a part of the multisensory platform for SHM of cultural heritage objects, which will then allow to georeference the results of measurements performed by this device and apply corrections to observations acquired by the sensor. This is a crucial element required for further data processing, essential for the analyses related to the object structure and its features and allowing spatial and multitemporal analyses.

This article is composed of several parts, including a short review of available solutions for indoor positioning (Section 2) together with the description of applied orientation model and characteristics of measuring and geolocalisation techniques used in performed experiments—classical surveying, terrestrial laser scanning, and close-range photogrammetry. In Section 3, a full and detailed description of the proposed methodology was presented. The results of performed experiments together with their brief analysis and interpretation are the subject of the Section 4. Section 5 contains a comprehensive analysis and discussion concerning obtained results and provides the conclusion of work performed.

## 2. Review of Indoor Geolocalisation Methods

### 2.1. Indoor Positioning Systems

Indoor Positioning provides accurate information about the location of objects and people inside the measured structure [16]. In the recent years, the interest in indoor localization has increased resulting in a rapid development of Indoor Positioning Systems (IPS). As opposed to the outdoor environment, where Global Navigation Satellite Systems (GNSS) are applied for localization and navigation, in case of indoor localization there is no standard system, which could be used on a mass scale.

**Figure 1:**
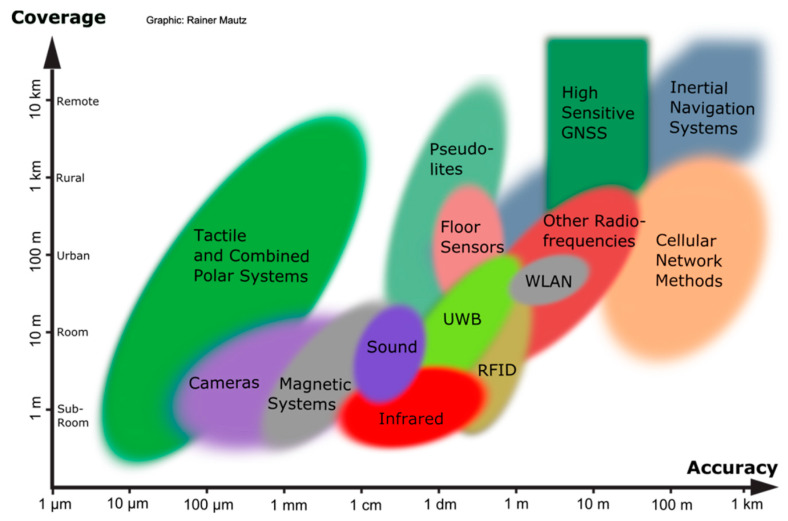
Overview of indoor technologies in dependence on accuracy and coverage [17].

Various technologies have been applied in the field of indoor positioning and navigation, which include techniques based on Radio Frequency (RF), ultrasound, audible sound, magnetic sensors, infrared (IR), and visual analysis-based systems. Mautz [17] presented an overview of indoor technologies along with their accuracy and coverage (Figure 1).

RF systems are further categorised into Bluetooth Low Energy (BLE), Ultra-wideband (UWB), ZigBee, Wireless Sensor Network (WSN), Wireless Local Area Network (WLAN), Radio-Frequency Identification (RFID), and Near Field Communication (NFC) [18]. Among these methods, WLAN, BLE and ZigBee are popular due to high availability of hardware. The accuracy of RF methods ranges from several meters (WLAN, BLE, ZigBee) [19,20], through several decimetres (UWB) [21,22,23,24,25] up to several centimetres (RFID) [23,26,27,28]. Numerous researchers investigated the usability of RF methods [24,29,30,31] in indoor positioning systems.

Ultrasound systems like the Cricket, Active Bat, and the Dolphin system [18,32] are also successfully applied in the field of IPS for being scalable, accurate (up to several centimetres), and low-cost [23,33]. However, these systems are sensitive to changes of environmental parameters like humidity and temperature [34].

Infrared positioning systems are generally exploited in three forms: active beacons, infrared imaging using natural radiation and artificial light sources [17]. This solution is low cost, but on the other hand, most IR systems require clear line-of-sight, which limits their usability in indoor positioning. Depending on a measuring principle accuracy ranges from several meters (Active Badges) to several centimetres while systems based on high resolution infrared sensors are able to detect artificial IR light sources at submillimetre accuracy [17].

Optical/Visual positioning systems involve determining the target position by identification of a marker or image, which is within view with the aid of a mobile sensor or a camera carried by the user [18]. Mautz [35] investigated different possibilities of reference data acquisition including reference acquired from 3D building models, images, deployed coded targets, projected targets, and observations performed by other sensors. For this task, simultaneous localization and mapping (SLAM) also can be applied [35], but it should be noticed that the use of these algorithms is not limited to vision-based methods only [36,37].

Techniques such as terrestrial laser scanning, photogrammetry, or classical surveying may also be considered as tools for optical/visual positioning since they exploit visual and optical observations in order to determine the position of a measured point. The main advantages of these methods are their low cost (especially in case of close-range photogrammetry), high accuracy (several millimetres and higher), widely available software, and known data processing workflow. For these reasons, their applicability as a tool for positioning of the proposed electromagnetic sensor was investigated in this work.

### 2.2. The Classical Surveying

In order to determine the position of a measuring device, it is necessary to perform the exterior orientation process resulting in the acquisition of so-called exterior orientation parameters, i.e., the location of the sensor body in the assumed reference system together with its rotation angles.

The basic geodetic method for calculating the position of a point in 3D space is tacheometry, which is based on the measurements of distance and angles [38]. Through a single measurement to a point its $X_{p},\;Y_{p},\;Z_{p}$ coordinates can be directly determined (Equation (1)):

(1) $$\begin{array}{c} X_{p}=X_{st}+d*\cos\left(Az\right)\\ Y_{p}=Y_{st}+d*\sin\left(Az\right)\\ Z_{p}=Z_{st}+d*\tan\left(\alpha_{v}\right), \end{array}$$

where $X_{st},\;Y_{st},\;Z_{st}$—known coordinates of total station position, d—horizontal distance, Az—azimuth to the measured point, $\alpha_{v}$—vertical angle.

The described method is burdened with some disadvantages such as relatively low accuracy (depending on the accuracy of the measuring equipment) and low reliability understood as the possibility to check the correctness of observations relatively to each other and to detect errors in measurements. In order to increase the accuracy of point position and its reliability, multiple angular and linear observations from several total station positions must be conducted. The establishment of geodetic network of points with interrelated geometric relations is also required. In this case, the number of observations is greater than the number of unknowns (the number of points multiplied by the number of coordinate components). The coordinates of points in the network are calculated by least squares adjustment. Teunissen [39] applied the well-known Gauss-Markow (Equation (2)) linear model (a linearized form of the input nonlinear relationships). This linear model is also used in the photogrammetric bundle adjustment process [40].

(2) $$Ax+e=y;\;\;\;e\;\sim \;\left(0,\;C_{e}\right)$$

where:

A—design matrix (*n* × *u*) (*n*—number of observational equations, *u*—number of unknowns), rank(A)=u (full rank);
x—parameter vector (*u* × 1);
e—true error vector (*n* × 1);
y—observation vector (*n* × 1) (uncorrelated observations);
$C_{e}$—observation error covariance matrix (*n* × *n*) (positively determined), which is also the observation result covariance matrix, i.e., $C_{e}\equiv C_{y}$;

As a result of the above equation, the final coordinates of points are calculated together with the accuracy characteristics. It is worth noting that this particular method provides full knowledge about the accuracy of coordinate calculation, which can be derived from the covariance matrix. No other method ensures such complete characteristics of measurements accuracy.

### 2.3. Terrestrial Laser Scanning

Terrestrial Laser Scanning (TLS) is a range-based measuring technique [41], which has gained much popularity in a variety of fields including industry, civil engineering, damage assessment and deformation monitoring, forestry inventory or cultural heritage documentation [42,43,44,45]. As TLS is based on a laser beam deflection, it allows for the acquisition of large sets of accurate 3D metric data automatically and in a short time.

The result of such measurement is a 3D point cloud representing the shape of measured objects together with the intensity of the returning signal which may be successfully applied in examination of properties of investigated objects [46] such as their reflectance, roughness, moisture, brightness, and grain size [47,48]. However, in the case of geolocalisation purposes, the significant factors are measurement accuracy and resolution. 

The accuracy of TLS measurements ranges from several centimetres up to submillimetre-level [49,50], however, it depends on many factors including the environment conditions, properties of a tested object, instrumental errors [51,52,53], as well as scanning geometry [54]. Some of them can be eliminated or minimised either by applying corrections to measured angles and distances or using the system’s calibration [55,56]. However, environment conditions such as inconvenient temperature, rain or dust are difficult to predict and model. Furthermore, the reflection intensity has an effect on the accuracy of range measurements. Voegtle [47] investigated the influence of different materials on TLS measurement accuracy and proved that for surfaces of brighter colour (and consequently higher intensity) the geometric accuracy was about 3 times higher compared to black surfaces (of low intensity). Moreover, in the case of partially transparent materials and specular surfaces, the geometric accuracy decreases rapidly [47,57,58,59,60].

The measurement resolution is determined by an angle increment, the incidence angle, scanning distance, and spot size called beam divergence [61,62]. The size of the laser spot (called divergence) increases with the increasing scanning distance affecting the accuracy of point position [63] and decreasing the scanning resolution. Moreover, beam divergence is associated with the “mixed edge” problem meaning that a part of the beam is reflected by the object’s edge, but the other part travels further and is reflected later by another object. In consequence, there is more than one distance recorded for a single signal, the final distance value, which is determined as a mean distance, can be inaccurate [61].

### 2.4. The Structure-from-Motion/Close-Range Photogrammetry

Close-range photogrammetry is another measuring technique for digital 3D reconstruction of the objects’ shape. Unlike terrestrial laser scanning, which is an active technique, photogrammetry is an image-based passive method, which provides high resolution and accurate datasets, being at the same time a low-cost solution characterized by high level of automation. These features made close-range photogrammetry very flexible and thus, widely used measuring method successfully applied in a variety of fields including civil engineering [64,65], forestry [66,67], geology [68,69], archaeology [70,71], and cultural heritage documentation [72,73].

Modern close-range photogrammetry applies methods and algorithms are derived from Computer Vision (CV) such as Structure-from-Motion (SfM) and Multi-View Stereo (MVS). The main function of SfM is to calculate the image orientation based on a series of overlapping, offset images [74], while MVS is used for dense point cloud generation. SfM is based on two principles: the binocular vision and the changing vision of the object, which is moving or being observed from different points of view [66]. 

As the parameters of internal orientation of the camera are calculated via self-calibration, the use of consumer-grade non-metric cameras is possible. Camera positions and scene geometry are reconstructed simultaneously during bundle adjustment, which involves image matching (the process of detecting corresponding features on multiple images). Image matching algorithms usually perform poorly in the case of objects and scenes where corresponding (homologuous) features cannot be clearly identified, so for areas characterized by poor or no texture as well as repetitive patterns. Apart from texture quality, several other factors influence the accuracy of image orientation: the base-to-depth (B/D) ratio, the number of measured tie points per image during image matching, the number of images, on which a certain tie point is visible, and the accuracy of self-calibration of the camera [75].

Camera positions and 3D scene derived from SfM lack scale and orientation in a specific coordinate system. To georeference them in a particular coordinate system (e.g., geodetic coordinate system), it is possible to apply 7-parameter transformation based on ground-control points with known coordinates [76]. In case of the objects, the shape of which must be accurately reconstructed but their position in space is irrelevant, scale bars with known length may be applied instead. Known image orientation and intrinsic camera parameters calculated by SfM allows the generation of a dense point cloud, and for this purpose, the MVS algorithms are usually used [77]. Resulting point cloud can be further processed for 3D modelling and othophoto generation.

Similar to other techniques, close-range photogrammetry has some limitations resulting from applied algorithms and methods. However, today the integration of several complementary techniques is becoming more and more popular as it allows for the enhancement of the final results.

### 2.5. The Rigid-Body Orientation

**Figure 2:**
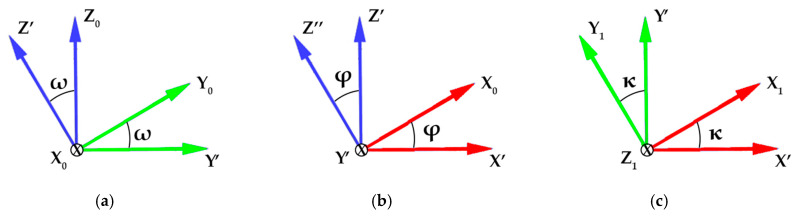
Three rotations about the coordinate axes; (**a**) primary rotation ω about X_0_—axis, (**b**) secondary rotation ϕ about Y’—axis, and (**c**) tertiary rotation κ about Z_1_—axis [41].

The main subject of this work was to examine different approaches aiming at the determination of exterior orientation parameters of the electromagnetic sensor at each sensor station. For this purpose, the sensor was treated as a rigid body, for which position and rotation angles had to be found in a defined reference system (Figure 2). In the performed experiments, the reference system was associated with the tested plane formed by walls to be examined by the electromagnetic sensor at each test site.

In order to determine 3 linear and 3 angular exterior orientation parameters, the 3D affine transformation is commonly applied. The transformation is performed using a minimum of three tie points, but if the number of points increases, the redundancy increases too, resulting in higher accuracy of data registration. This also allows the elimination of outliers and therefore provides better fitting of the 3D transformation model into performed observations.

The relation between the sensor system and the global reference system is expressed in Equations (3) and (4) [78,79,80].

(3) $$M_{ext}=R_{\omega \varphi \kappa }*M_{int}+T,$$

(4) $$R_{\omega \varphi \kappa }=R_{1}\left(\omega \right)\;R_{2}\left(\varphi \right)\;R_{3}\left(\kappa \right),$$

where $M_{ext}$ is the vector of point coordinates in the global system,$\;R_{\omega \varphi \kappa }$ is the rotation matrix, $M_{int}$ is the vector of point coordinates in the local (sensor) system, T is the translation matrix, $R_{1}\left(\omega \right)$ is the rotation matrix in relation to the x-axis by angle ω*,* $R_{2}\left(\varphi \right)$ is the rotation matrix in relation to the y-axis by an angle φ, and $R_{3}\left(\kappa \right)$ is the rotation matrix in relation to the z-axis by an angle κ. The final representation of the rotation matrix [81]$\;R_{\omega \varphi \kappa }$ related to the each of angle can be expressed as Equation (5)

(5) $$R_{\omega \varphi \kappa }=\left[\begin{array}{ccc} cos\;\varphi \;cos\;\kappa & -cos\;\varphi \;sin\;\kappa & sin\;\varphi \\ cos\;\omega \;sin\;\kappa \;+\;sin\;\omega \;sin\;\varphi \;cos\;\kappa & cos\;\omega \;cos\;\kappa \;-sin\;\omega \;sin\;\varphi \;sin\;\kappa & -sin\;\omega \;cos\;\varphi \\ sin\;\omega \;sin\;\kappa -cos\;\omega \;sin\;\varphi \;cos\;\kappa & sin\;\omega \;cos\;\kappa \;+\;cos\;\omega \;sin\;\varphi \;sin\;\kappa & cos\;\omega \;cos\;\varphi \end{array}\right],$$

This representation of rotation and translation allows the independent orientation of the sensor system in a global, external reference system. However, in order to perform structural health monitoring, it is necessary to obtain the relative orientation i.e., the relation between the examined wall and the face of the sensor system. To compute this relation, it is necessary to: firstly, define a new reference system related to the wall, secondly, transform the coordinates of all points into the new coordinate system and finally, calculate the sensor’s translation and rotation parameters in the new coordinate system. In this case, it is not possible to directly implement the rotation matrix presented in the Equation (5) because the values of the rotation angles ω,φ,κ need to be found first. To do this, the relations between normal vectors of the reference plane and the sensor plane was computed.

**Figure 3:**
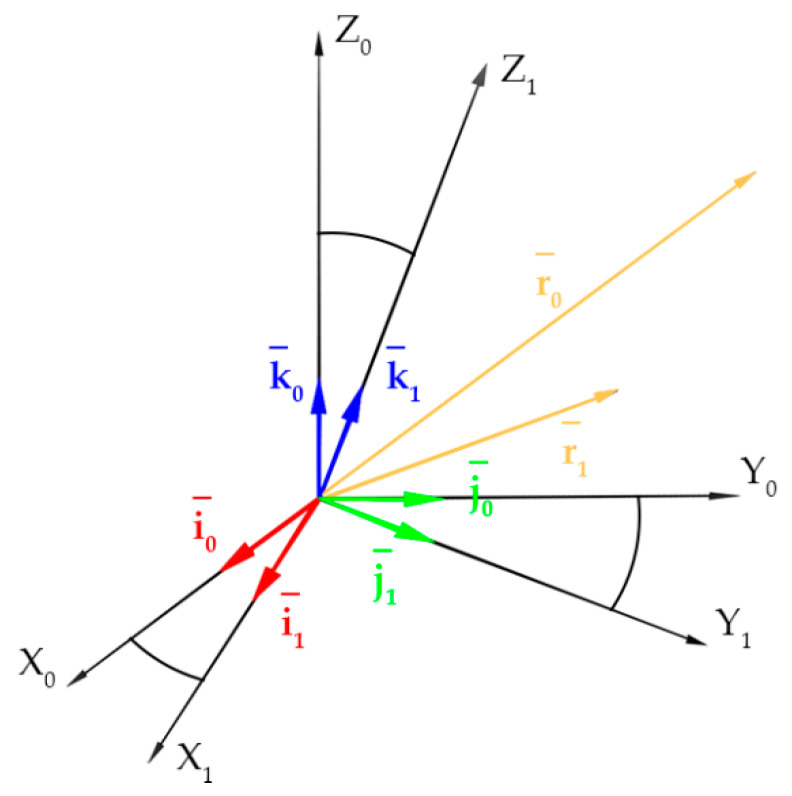
The coordinate system related to the vector r.

To describe the rotations [82], the $O\;x_{0},\;y_{0},\;z_{0}$ coordinate system was used, followed by the vector r (Figure 3), to which the $O\;x_{1},\;y_{1},\;z_{1}$ coordinate system was associated. It is necessary to consider the relations, which occur between the coordinates of the vector r in the coordinate system $O\;x_{1},\;y_{1},\;z_{1}$ and the coordinates of this vector in the fixed (non-rotated) reference system $O\;x_{0},\;y_{0},\;z_{0}$. The position of the vector r before rotation is denoted by $r_{0}$, whereas by $\left\{i_{0},\;j_{0},\;k_{0}\right\}$ the standard orthogonal base in the system $O\;x_{0},\;y_{0},\;z_{0}$ is designated.$\;i_{0},\;j_{0},\;k_{0}$ are unit vectors along the $x_{0},\;y_{0},\;z_{0}$ axes, respectively. Similarly, $\left\{i_{1},\;j_{1},\;k_{1}\right\}$ is a standard orthogonal base in the $O\;x_{1},\;y_{1},\;z_{1}$ system.

The vector r is presented in the system $O\;x_{0},\;y_{0},\;z_{0}$ as follows:

(6) $$r_{0}=x_{0}i_{0}+y_{0}j_{0}+z_{0}k_{0},$$

However, the same vector r is presented in the system $O\;x_{1},\;y_{1},\;z_{1}$ as follows:

(7) $$r_{1}=x_{1}i_{1}+y_{1}j_{1}+z_{1}k_{1},$$

The r vector is represented by the $r_{0}$ and $r_{1}$ vectors, so the relationships between the components of the r vector in both coordinate systems are:

(8) $$r_{0,x}=r_{0}i_{0}=r_{1}i_{0}=x_{1}i_{1}i_{0}+y_{1}j_{1}i_{0}+z_{1}k_{1}i_{0},$$

Analogous formulas were obtained for the components $r_{0,y}$ and $r_{0,z}$ with:

(9) $$r_{0,y}=x_{1}i_{1}j_{0}+y_{1}j_{1}j_{0}+z_{1}k_{1}j_{0},$$

(10) $$r_{0,z}=x_{1}i_{1}k_{0}+y_{1}j_{1}k_{0}+z_{1}k_{1}k_{0},$$

The Equations (8)–(10) are presented in the form of a vector equation as follows:

(11) $$r_{0}=R_{1,0}r_{1},$$

The Matrix $R_{1,0}$ represents the transformation of coordinates of the point P from the system $Ox_{1},\;y_{1},\;z_{1}$ to the system $Ox_{0},\;y_{0},\;z_{0}$ and it is written in the form:

(12) $$R_{1,0}=\left[\begin{array}{ccc} i_{1}i_{0} & j_{1}i_{0} & k_{1}i_{0}\\ i_{1}j_{0} & j_{1}j_{0} & k_{1}j_{0}\\ i_{1}k_{0} & j_{1}k_{0} & k_{1}k_{0} \end{array}\right],$$

The coefficient values in the Equation (12) enable to calculate the rotation angles ω,φ,κ of the sensor plane relative to the wall plane described in the Equation (5). In this particular case, the unit vectors $i_{0},\;j_{0},\;k_{0}$ and $i_{1},\;j_{1},\;k_{1}$ are the parameters of the normal vector of the wall plane and the sensor plane, respectively.

In order to determine the plane equation parameters minimum three points are needed. The vector product r for two vectors $r_{1}$ and $r_{2}$ formed according to the Equation (13)

(13) $$r=r_{1}\times r_{2},$$

As the number of control points is four or more, the least square adjustment [83] method is used to compute the parameters [a,b,c] from the Equation (14).

(14) $$a\left(x-x_{0}\right)+b\left(y-y_{0}\right)+c\left(z-z_{0}\right)=0,$$

Since the examined object is represented by a very large number of points (e.g., a cloud of points from laser scanning or the SfM method), the pcfitplane [84] function in the Matlab software was used for the calculation. The plane is described by a planeModel object while maxDistance is a maximum allowed distance from an inlier point to the plane. Plane parameters are specified as a 1-by-4 vector. The four parameters [a, b, c, d] describe the equation for a plane: ax+by+cz+d=0. This function uses the M-estimator SAmple Consensus (MSAC) algorithm to find the plane [85].

The last element is the calculation of the distance d between the point ($x_{0},\;y_{0},\;z_{0}$), which is the center of the sensor and the wall plane [a,b,c]. The Formula (15) was used:

(15) $$d=\frac{\left|ax_{0}+by_{0}+cz_{0}\right|}{\sqrt{a^{2}+b^{2}+c^{2}}}\;,$$

## 3. Materials and Methods

Structural health monitoring (SHM) involves the use of numerous instruments, i.e., sensors and other measuring devices that provide the measurement and assessment of features and parameters characterising investigated objects. There are many ready-to-use solutions available on the market for SHM, however, in case of monumental objects, which require non-invasive, contactless SHM methods, there is a need for development of novel measuring techniques, which are capable of detecting previously unknown features/conditions of the objects. This article concerns preparatory works that would be required prior to the use of data acquired by an electromagnetic sensor applied for SHM purposes.

**Figure 4:**
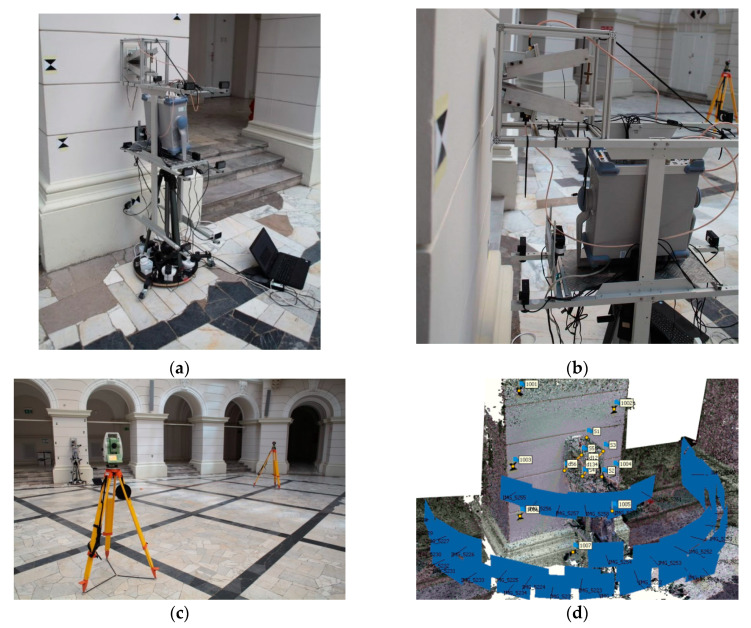
Setup of the proposed sensor system: (**a**) back view—electromagnetic (EM) sensor mounted on a special tripod with Vector Network Analyzer (VNA) Rohde and Schwarz ZVL13, (**b**) side view—EM sensor mounted in a wireframe box with VNA and four low-cost cameras (not used in this investigation), (**c**) the example of a total-station (TLS) position used for control/check points determination, and (**d**) the visualisation of the full-frame camera Canon EOS 5D Mark II positions.

The multisensor platform (Figure 4) is a prototype instrument consisting of an electromagnetic sensor and four digital cameras installed on a metal frame, full-frame camera Canon EOS 5D Mark II and Terrestrial Laser Scanner Z + F 5006 h. The proposed electromagnetic (EM) sensor system consists of two wideband horn antennas that operate in the frequency range of 2–18 GHz. The antennas are attached to the designed aluminum frame at a certain angle to each other in order to control the penetration depth of the sensor signal. Both antennas play different role in the setup, namely one acts as a transmitter and the second one acts as a receiver of the reflected signal. The measurements are provided using S-parameters, namely S_21_ (transmitting the signal from port one and receiving to port 2) via Vector Network Analyzer (VNA) Rohde and Schwarz ZVL13. The captured data is in a complex data format (real and imaginary data), which is recorded using a bespoke LabVIEW program on a laptop that is connected to the VNA via TCP/IP connection. The sensor system is positioned 2–6 cm from the measured object to avoid the contact, i.e., the measurements are contactless. This approach is a primary requirement when it comes to measurements of cultural heritage objects/structures to avoid any potential damages to their surface.

It must be stressed that the results of measurements performed by the electromagnetic sensor are not the subject of this study. The aim of this research is to propose and examine different methods of georeferencing of the sensor system to apply corrections to the sensor readings at further stages of data processing. The results of performed experiments will establish the data processing workflow, which will be a basis for future work.

### 3.1. Overview of the Approach

**Figure 5:**
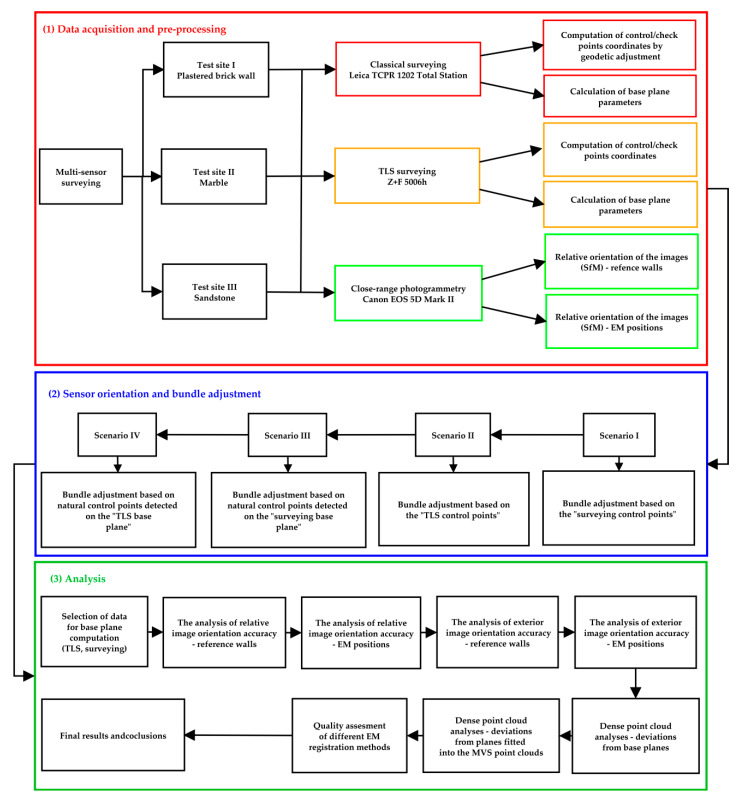
Diagram of the performed experiments: data acquisition, processing, and analysis.

The proposed method of geolocalisation of the sensor system is a multi-stage process based on the combination of commonly used algorithms implemented in Agisoft Metashape and the original software. The proposed method is a novel approach allowing both orientation of the electromagnetic device and its integration with other measuring devices. The whole experimentation involved the acquisition of data on three test sites differing in features, containing materials and surface types commonly present in case of cultural heritage objects. Detailed characteristics of the test sites were presented in the next section. Figure 5 illustrates the scheme of research work and experiments performed.

The process of sensor registration is an original approach resulting in determination of exterior orientation parameters (the XYZ coordinates of the antenna centre and three angular parameters—ω,φ,κ) of the EM sensor as well as relative orientation parameters in relation to the reference planewall, which is a subject of measurement performed by the electromagnetic sensor at each test site. In this work the plane corresponding to the examined wall is called “the base plane”. Calculation of these parameters (performed in two different ways) makes it possible to determine the sensor position, but on the other hand they can also be used in the EM data processing as a basis for the calculation of corrections applied to the raw data acquired by the sensor.

The whole experimentation was divided into three main parts: (1) data acquisition and pre-processing, (2) a set of tests concerning sensor orientation methods, and (3) the analysis of obtained results.

The process of data registration presented in this paper consists of the following stages:

1. Determination of the reference system and base planes (data pre-processing).

At this stage the reference system was established and the data serving as reference for further analyses were determined.

1.1. Three different surveying methods were selected for this purpose: classical surveying with Leica TCPR 1202 Total Station (TS), terrestrial laser scanning (TLS) with a Z + F 5006 h terrestrial laser scanner, and close-range photogrammetry (SfM/MVS method) with a Canon EOS 5D Mark II SLR camera.
1.2. Data processing at this stage involved the computation of coordinates of control points and check points as well as the determination of parameters of base planes at each test site. For these two operations, two sources of data were used: total station observations and TLS measurements. Both geodetic measurements and TLS data were processed separately. As a result, two sets of point coordinates and two base planes (TS base plane and TLS base plane) were calculated for each test site.
1.3. The coordinates of control points and check points were calculated separately in two ways: based of TS observations through geodetic adjustment and based on TLS measurements, from, which the XYZ values for each point were derived.
1.4. The parameters of base planes were computed using the coordinates of control points.
1.5. For photogrammetric elaboration of close-range images, the SfM approach was used. The proposed methodology involved the pre-processing step of verification whether the image sets were acquired correctly. For this purpose, relative orientation of images was performed separately for each dataset containing the images covering the wall at each test site (denoted as SfM—the wall on the diagram) as well as for sets of images taken at each sensor’s position (denoted as SfM—EM positions).

2. Sensor orientation involving the bundle adjustment.

For this purpose, four different scenarios for calculation of exterior orientation parameters of the sensor were proposed:

2.1. Scenario I: this scenario involved the use of marked control and check points during the bundle adjustment. Point coordinates were derived from classical surveying measurements. These points were measured both on the images covering only the wall and on the images containing both the sensor and the wall at each station.
2.2. Scenario II: similar to the first scenario, the second method involved the use of signalised control and check points in the bundle adjustment. However, in this case the coordinates of these points were derived from TLS measurements.
2.3. Scenario III: since the measurement of reference points is time-consuming, the possibility to reduce the time of measurement in field was tested in Scenario III. This scenario involved the use of a featured-based orientation method implemented in the Agisoft Methashape software. Initially the images covering only the wall were aligned with the use of control and check points measured with a total station. This set of images served as a reference in the next step of data processing. The next step involved the relative orientation of images taken at each sensor station. Sets of images covering single stations were aligned, so that their relative orientation was computed. Next, tie points were automatically detected both on the images of each wall and on the images of the sensor at each position. Based on these tie points, the exterior orientation of image sets of all sensor stations was calculated.
2.4. Scenario IV: the fourth scenario consisted of the same steps as Scenario III with the difference that in this case the coordinates of signalised points were derived from TLS data.

3. The analysis of results obtained with each scenario.

Each of the above-mentioned geolocalization scenarios was examined with regard for their accuracy and efficiency. Efficiency in this case shall be understood as the time which is necessary to measure reference points and then calculate their coordinates applying different scenarios. Complete analysis required the verification of parameters listed below:

3.1. The verification of base plane approximation using different source data: as the orientation of the EM sensor is computed in relation with the base plane, firstly it is required to verify the accuracy of approximation of the base plane. Two datasets were tested as source data to approximate the base plane: TLS data and classical surveying data. The quality assessment contained: (1) the analysis of RMSE values obtained on marked control and check points measured with a total station, (2) the analysis of RMSE values obtained on marked control and check points measured by TLS, and (3) the analysis of deviations between the TLS base plane and measured TLS points.
3.2. The analysis of relative image orientation accuracy performed both for “the wall” and “the wall + the sensor” datasets on each test site: in order to check whether images were properly acquired and also to verify the correctness of their relative orientation, the inspection of RMSE reprojection errors appearing on natural and signalised control/check points was performed.
3.3. The analysis of exterior image orientation using RMSE values obtained on control and check points performed both for “the wall” and “the wall + the sensor” datasets on each test site: in order to verify the quality of exterior orientation of the images, the quality assessment and accuracy analysis concerning marked control and check points were performed for each test site.
3.4. The analysis of exterior image orientation using scalebars: additional verification involved the accuracy assessment on scalebars located both on the wall and on the sensor frame.
3.5. Dense point cloud analysis involving base planes: it consisted of the calculation of deviations of dense point clouds generated for all sensor stations, obtained in various scenarios from the base planes determined at earlier stages of data processing. The purpose of this analysis was to check the possible quality of the approximation of a reference plane based on the MVS point cloud (not the control points). Tests included the analysis of deviations from the TS base plane calculated for the dense point clouds generated for Scenarios I and III and as well as the deviations from the TLS base plane observed for the dense point clouds generated for Scenarios II and IV.
3.6. Dense point cloud analysis involving point cloud sampling—the aim of the analysis was to find the optimal subsampling resolution for plane detection; for this purpose, dense point clouds generated for all sensor stations were subsampled with four resolutions (5 mm, 10 mm, 30 mm, and 50 mm). Then, for each of them the deviations from the MVS reference plane was examined.
3.7. The analysis of skews between base planes and reference planes fitted into dense point clouds generated for each sensor station and the assessment of ω,φ,κ angles of the sensor plane.

The results of analyses described above made it possible to assess which methods of data registration allowed to obtain the highest accuracy and efficiency.

### 3.2. Overview of Source Data and Selected Test Sites

**Figure 6:**
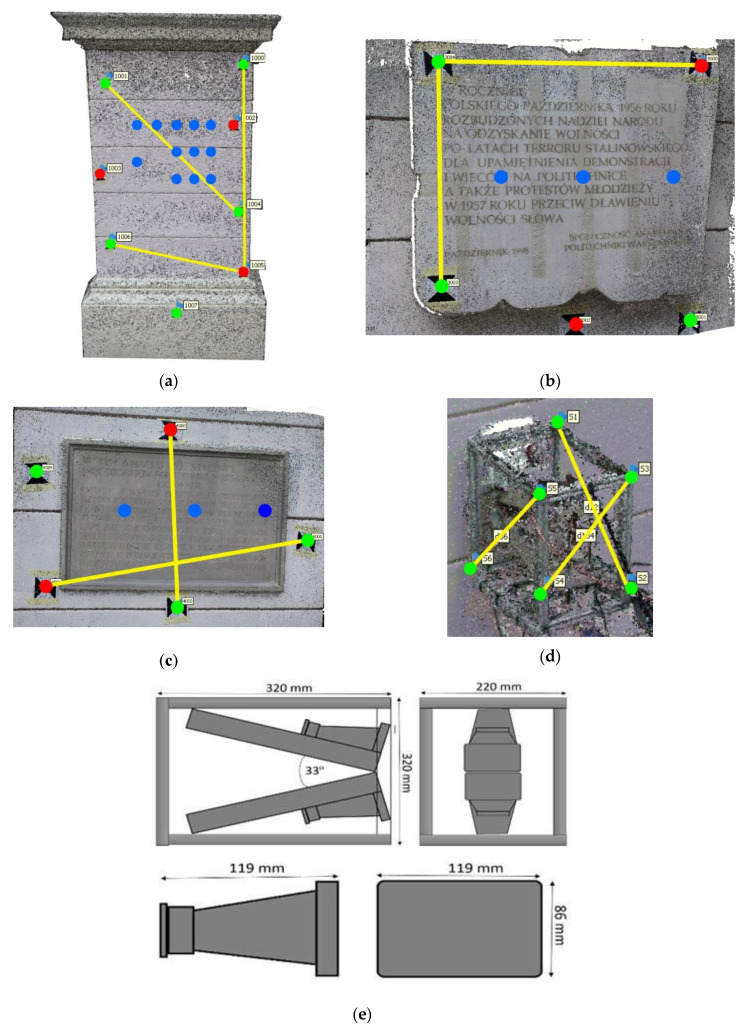
Dense point clouds generated for each test site: (**a**) Test site I—the plastered brick wall, (**b**) Test site II—marble plaque, (**c**) Test site III—sandstone plaque, (**d**) sensor with marked scalebars and check points, (**e**) the dimensions of the horn antenna; marked reference points are visible (black and white crosses); control points (green dots), check points (red dots), EM sensor positions (blue dots), and scale bars (yellow lines).

In order to examine each of the proposed methods for EM sensor registration, three test sites were established: a fragment of a plastered brick wall and two memorial tables: one made of marble and one made of sandstone. All test sites were located in the Main Hall at Warsaw University of Technology, Warsaw, Poland. Figure 6 presents these test sites along with the sensor positions and locations of control/check points and scalebars.

Test site I covered a fragment of a plastered brick wall, where 12 EM sensor positions (blue dots) were established (Figure 6a). For each position, approximately 25 images were taken with an SLR Canon camera. From the set of reference points marked on this site, 5 control points and 3 check points were defined. Test site II (Figure 6b) covered a marble memorial table, while test site III was a memorial table made of sandstone. In both cases 3 control points, 2 check points, and 3 sensor positions were established. For each of these two test fields approximately 20 images were acquired. As the sites both covered a very small area, it was impossible to establish more than 3 control points and 2 check points.

The coordinates of control and check points were obtained with the use of two measurement techniques: a total station survey (geodetic surveying) and terrestrial laser scanning. The geodetic measurements were performed using Leica TCRP 1202 total station, which provides the angular accuracy of 2”, the accuracy of reflector distance measurement of 2 mm + 2 ppm and the accuracy of reflectorless distance measurement of 3 mm + 2 ppm. TLS measurements were done with a Z + F Imager 5006 h terrestrial laser scanner. In the case of total station measurements, the point coordinates were calculated by means of geodetic adjustment while in the case of TLS data a semi-automatic calculation method implemented in Z + F LaserControl software was applied. Table 1 presents the accuracy of coordinates of the reference points expressed through RMSE values.

The accuracy of point coordinates is presented in a different way depending on the applied measurement technique because of different methods used to calculate them.

Surveying involves a direct measurement of a distance from the instrument to the desired point as well as and two angles: horizontal and vertical. The final XYZ coordinates are computed in the process of geodetic adjustment. On the contrary, TLS technique does not allow direct measurements to selected points, but the surrounding areas are scanned. For this reason, it was necessary to use the algorithm implemented in Z + F LaserControl software, which identifies the center of the target automatically based on the initial part of the point cloud target area. Therefore, the accuracy of coordinates is represented by linear RMSE without splitting it into XYZ components.

The RMSE values presented in the Table 1 indicate similar accuracy of results obtained by TLS measurements on all test sites. However, this trend cannot be observed for total station measurements—it is clearly seen that RMSE values observed for the test site I differ significantly from the others. It is a consequence of applied measurement methodology as well as resulting accuracy of the adjustment of observations. Unlike the test sites II and III, where only one measurement station was established, in case of the test site I there were 3 instrument stations. Increased number of stations resulted in higher accuracy of the adjustment process as it was possible to include angular measurements, which are characterized by higher accuracy than distance measurements. In the case of test sites II and III, only one instrument position was used so it was necessary to include the distance measurement in the adjustment process which affected the final accuracy of reference points. These values were used as weights in the final SfM bundle adjustment process.

## 4. Results and Discussions

### 4.1. Selection of Data for Base Plane Computation

In order to calculate the exterior orientation (EO) parameters of the EM sensor, a set of reference points was used. For this reason, the accuracy of their coordinates was one of the key factors affecting the accuracy of EO parameters. Moreover, further stages of data processing required the determination of the sensor orientation relative to the base plane, which was determined based on the reference points. For these reasons, it was necessary to consider which measurement method should be applied and which configuration of points should be used in the process of determination of the base plane (Table 2). For the accuracy analysis, two types of points forming a common plane were used: control points—in order to calculate the plane parameters—and check points used for an independent accuracy analysis. It should be stressed that control and check points only for the Test Site II are different than those used in the image bundle adjustment process, due to the possibility of marking points on the historical marble. Additionally, in the case of the TLS data, for each point the distance (deviation) between the point and the base plane was calculated. The Table 2 presents the number of used control and check points as well as the results of quality assessment of base plane determination.

Based on the residuals presented in Table 2, it may be stated that for test sites, on which measured objects were made from different materials, only the use of marked points provides high orientation accuracy. The crucial aspect to consider is whether a particular measuring technique is appropriate to measure objects made of a certain material. TLS measurements are known for achieving low accuracy while used to scan transparent surfaces. For this reason, it is not recommended to use TLS point cloud for plane fitting in the case of test site II, where semi translucent marble was a dominant material.

### 4.2. Relative Orientation of the Images Covering the Walls

**Figure 7:**
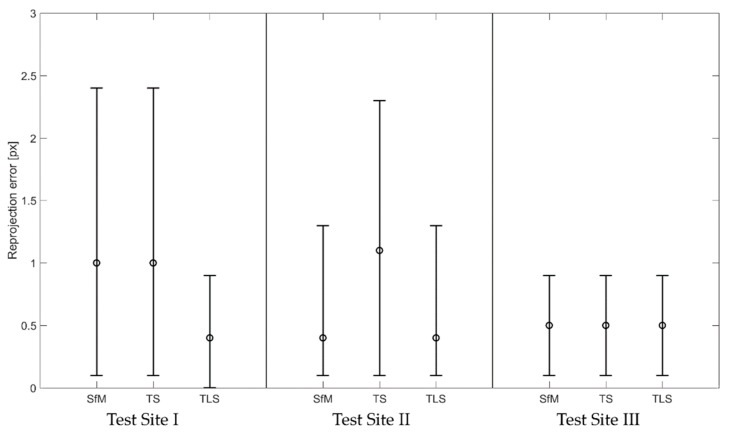
The sketch of the reprojection errors with mean (marked as circles), maximum and minimum values on marked points for three test sites for Structure form Motion method: SfM—without coordinates of the reference points, TS—total station method, and TLS—TLS method.

For the calculation of relative camera orientation, three approaches were applied. (1) The “SfM” method involved the use of signalised points as manually determined tie points during the bundle adjustment, but there were no coordinates in any external coordinate system assigned to them—the resulting model had no scale. (2) In the "TS” method point coordinates were determined by geodetic measurement with a total station. (3) In the "TLS” method point coordinates were derived from TLS measurements (Figure 7). The accuracy of relative orientation was examined by the analysis of the reprojection error values, which is the basic indicator allowing to verify the correctness of a photogrammetric model.

Eight signalised points were used in the Test Site I and five signalised points were used for each Test Sites II and III. For each method, the coordinate system was defined in a different way, so the Figure 7 presents the linear values of reprojection error as they are independent of the coordinate system definition, therefore making the acquired results comparable.

Analysing the results presented in Figure 7, it can be concluded that in all cases the relative orientation has been calculated correctly as the mean RMSE of the reprojection error only in one case exceeds 1 pixel. However, it can be easily seen that RMSE values of the reprojection error obtained for Test Site I differ significantly depending on the applied method. In this case the crucial factor, which resulted in such effect, was the material and texture of the tested object. The Test Site I consisted of a plain wall with solid, uniform texture, which caused the decrease in number and quality of automatically detected key points used as tie points in the process of image matching. In case of the first test field, the highest relative orientation accuracy was obtained by the TLS method, while the remaining methods provided the same and slightly worse accuracy. It can be observed that including TLS control points during photo alignment improved the relative orientation quality and decreased the reprojection error.

The results obtained for the second test site indicate similar accuracy of the SfM and TLS methods and moreover, in this case they provided higher accuracy than the TS method. As far as the mean value of the RMSE of the reprojection error is concerned, it can be seen that its value is approximately 0.3 pixels while the maximum is about 1.2 pixels both for the SfM and TLS methods. These results suggest that unlike in the Test Site I, TLS reference points did not influence the quality of relative orientation.

It is noteworthy that the values of minimum, maximum and mean reprojection error are all the same for all methods in the case of the sandstone block. Summarizing the results obtained for all datasets, it can be observed that among all methods the highest accuracy was acquired by the TLS method, while geodetic measurements proved to slightly decrease the accuracy of relative orientation. When the additional control and check points are used, it is necessary to consider whether the quality of these points (meaning location accuracy and the accuracy of measurement performed independently with another measuring technique) do not have a negative impact on the final accuracy of the bundle adjustment. Accordingly, the analysis of results acquired in the relative orientation process should be analysed along with the results of exterior orientation. The errors obtained on control points should be taken into consideration in order to verify the correctness of the assumed mathematical model. Last, but not least, the analysis of errors calculated for check points would serve as an independent verification of obtained results.

### 4.3. Relative Orientation of the Images Covering Fragments of Walls and the Sensor

The second dataset consisted of images covering both reference walls and the sensor at each station. The Table 3, Table 4 and Table 5 contain the main characteristics of relative orientation accuracy obtained on three test sites. In this case, five different orientation methods were applied. Being aware of the principles of SfM algorithms, their method of operation and the way the object’s features influence the final result, it was supposed that RMSE values obtained for this dataset would be slightly lower than in the case of reference walls. This assumption was supported by the fact that the texture of pictures covering both the walls and the sensor is less uniform, and therefore, it allows to achieve better results at the stage of image matching. This assumption proved to be true on the Test Site I and in most cases on the Test Site II.

In order to verify if the images are correctly oriented, the errors were calculated separately for points located on walls (“Wall”) and points on the sensor frame (“Sensor”). Detailed description of applied orientation methods was presented in Section 3.1.

In Table 3, due to the 12 EM positions, authors decided to show only 3 positions with minimum, maximum, and mean linear values of the reprojection error. The values acquired for remaining stations are similar. The reprojection error values (Table 3) obtained for all methods and all sensor positions on the Test Site I indicate that in all cases the relative orientation was calculated correctly since the mean RMSE of reprojection error does not exceed 1 pixel. It is readily seen the results acquired by different orientation methods are very similar. It also should be noticed that in all cases the errors observed on points located on the wall are higher than these obtained for the points on the sensor frame, however these differences are small and may be neglected.

Reprojection error values obtained for Test Site II (Table 4) imply that high accuracy of relative orientation was achieved. It can be noticed that similar to the results obtained on the previous test site the reprojection error takes similar values for all orientation methods. However, the results obtained for Scenario I clearly stand out from the rest—in this case the reprojection error values are higher for all sensor positions. Mean reprojection errors observed on points located on the wall and points located on the sensor frame are either equal or very similar.

Once again acquired results, presented in Table 5, proved the correctness of relative orientation determination as the mean reprojection error varies between 0.4–1.3 pix, which are acceptable values indicating high relative orientation accuracy. It can be observed that in this test site the highest orientation accuracy was obtained by applying the Scenario I. Moreover, this method provided the smallest differences between points located on the wall and points on the sensor frame. It is also worth noting that for the Scenario II the difference between reprojection errors calculated for point located on the wall and the point located on the sensor frame is highest.

On the grounds of the above-described tests performed on three test fields it has been observed that at the stage of calculation of relative orientation of images the choice of orientation method is not crucial for obtaining the highest accuracy since the differences observed for different methods are rather subtle. Furthermore, at this stage also the impact of material type on the orientation accuracy was not observed.

### 4.4. Exterior Orientation of the Images Covering the Walls

In the next step of data processing, exterior orientation parameters for all cameras were calculated. All images were divided into the same datasets as at the previous stage of relative orientation. To obtain exterior orientation parameters, two methods were applied: TS method (Scenario I) and TLS method (Scenario II). These two approaches were described in detail in the Section 3.1.

**Figure 8:**
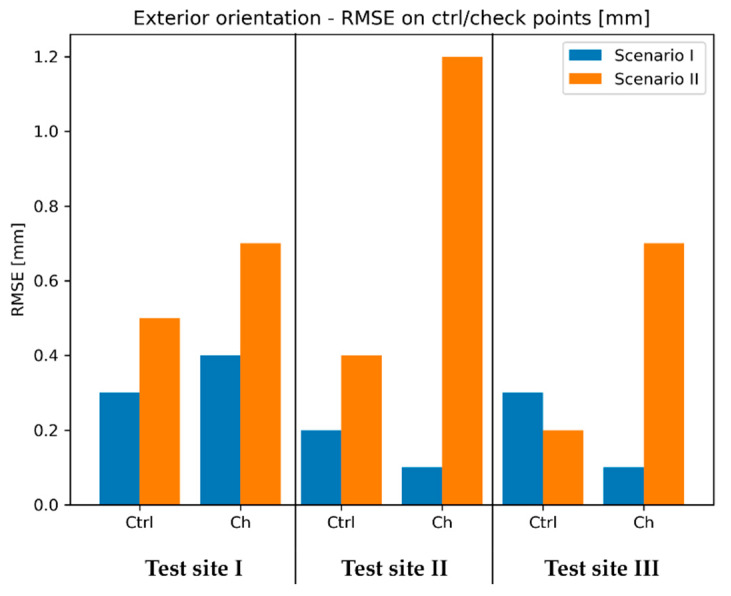
The graph presenting the RMSE values calculated for marked control/check points on three test sites.

Again, the first group of images, which was processed, was the dataset containing images of the walls (the sensor was not included). Exterior orientation of the images was determined with accuracy, the characteristics of which are presented in Figure 8.

The Figure 8 contains values of RMSE calculated using coordinates of signalized reference points, which were determined with the use of geodetic measurements and TLS data as explained in the Section 3.1. For additional control, the scale bar examination was introduced. For this purpose, three sets of scale bars were established on walls in each test field. The lengths of scale bars were calculated based on coordinates determined on models and compared with reference values derived from point coordinates measured on site with a total station. The scale bar parameter is as a mean of absolute values of differences between distances computed in the way described above.

In case of the Test Site I, both orientation methods provided submillimetre accuracy of the resulting model. However, analyzing RMSE values on reference points, it can be concluded that the Scenario I provided higher accuracy of exterior orientation determination. Nevertheless, mentioned difference in accuracy is rather subtle. Similarly, to the results obtained on the Test Site I, on the Test Site II the Scenario I also allowed higher accuracy of exterior orientation parameters determination. In contrast, in this case the difference in favour of the Scenario I is much more noticeable as both the RMSE of check points and the scale bar are about 3 times lower that in the TLS scenario. The accuracy of reference points was lower since terrestrial laser scanning has been proven to achieve low accuracy on semi-translucent materials (e.g., marble)—this resulted in lower accuracy of exterior orientation of the images. On the other hand, the tests performed on the Test Site III yielded different results that the previous two. In this case RMSE values obtained on check points indicate higher accuracy of the TLS scenario. However, the difference of scale bars is relatively small, suggesting similar internal consistency of both models.

### 4.5. Exterior Orientation of the Images Covering Fragments of Walls

**Figure 9:**
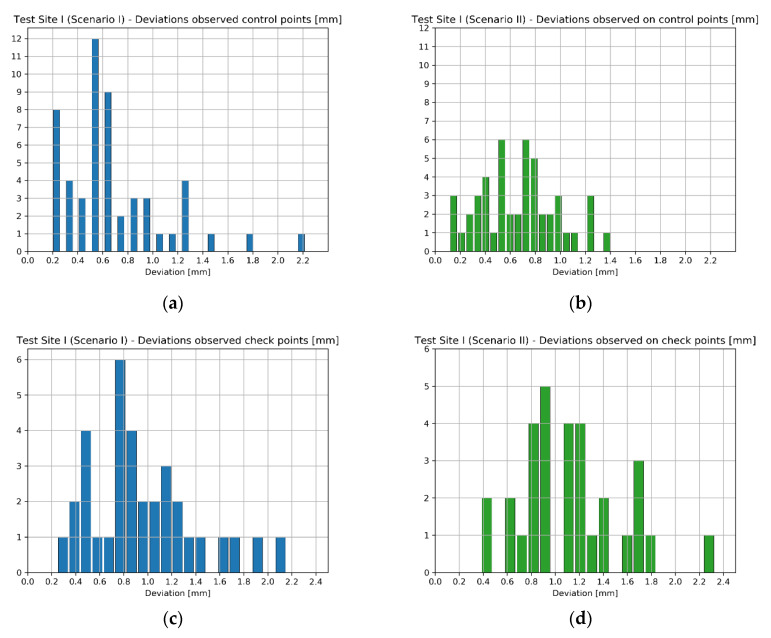

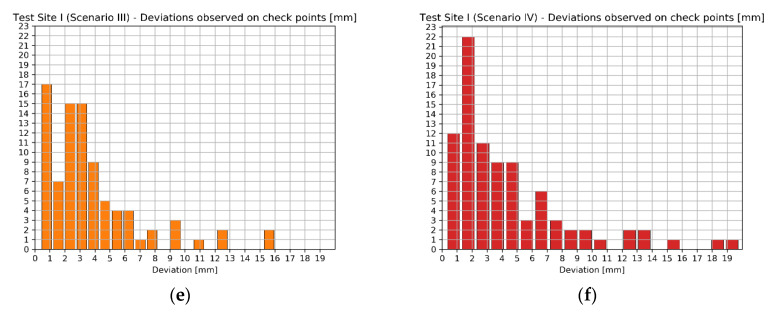
Test Site I: Histograms of deviations observed on control and check points (expressed in mm): (**a**) Control points classical surveying, (**b**) Control points TLS, (**c**) Check points classical surveying, (**d**) Checkpoints TLS, (**e**) Check points SfM with classical surveying, (**f**) Control points SfM with TLS for Test Site I.

**Figure 10:**
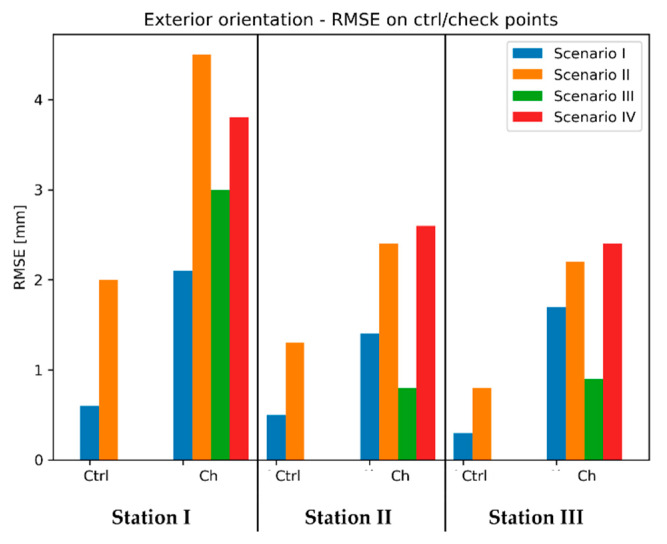
The sketch of the RMSE values on marked control/check points for Test Site II (Marble).

**Figure 11:**
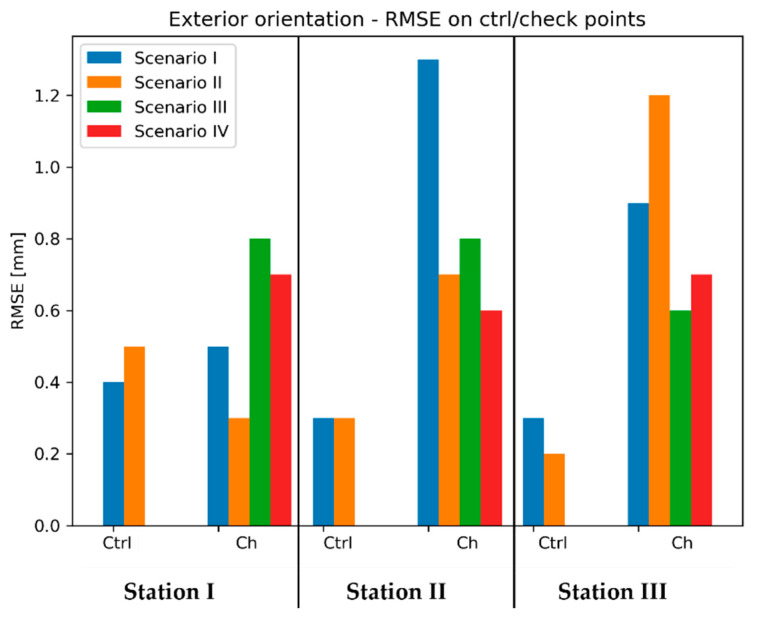
The sketch of the RMSE values on marked control/check points for Test Site II (Sandstone).

The next stage of data processing involved the determination of exterior orientation parameters of images covering the sensor together with fragments of scanned walls. According to the Figure 5 illustrating this phase, for this purpose four scenarios were applied. Figure 9, Figure 10 and Figure 11 contain the accuracy characteristics of exterior orientation of the images.

It can be noticed that RMSE values obtained on check points on all stations vary between 1 mm up to 3.9 mm and the RMSE on scale bars ranges between 0.3 mm to 4 mm. It should be noted that in all cases the accuracy achieved in the Scenario I is always higher than in Scenario III and analogically the situation is repeating with Scenario II and Scenario IV. Therefore, it can be concluded that on this test site the use of direct georeferencing with geodetic measurements and TLS reference data provides better results than 2-stage combined referencing involving SfM point-based approach. Moreover, in this case it cannot be stated clearly which one of these two combined scenarios offers better accuracy since for each of the three sensor stations the verdict should be different. In addition, Scenario I demonstrated slightly higher accuracy of exterior orientation. However, it should be noticed that the differences between these methods are relatively small, mainly in the difference of RMSE observed on scale bars.

The results obtained on the Test Site II lead to different conclusions than observations on the Test Site IV. First, it is clearly visible that both approaches engaging the use of TLS (Scenarios II and IV) provided lower accuracy than Scenarios I and III. The probable cause of this effect is due to the decreased accuracy of TLS measurements on semi-translucent materials, e.g., marble. Unlike the results obtained on the Test Site I, here the combination of TLS and SfM (Scenario IV) provided either higher (Station I) or similar (Station II and III) accuracy compared to the “direct” approach (Scenario II). This means that the use of this two-staged scenario may improve accuracy of orientation, which is decreased by other errors. Additionally, the use of combined approach “TS + SfM” (Scenario III) resulted in lower RMSE values of check points, suggesting higher positioning accuracy on Station II and III, while providing similar internal consistency of models being reflected by the scale bar value.

Compared to the results obtained on previous two test sites, the highest accuracy of exterior orientation was achieved on the Test Site III, which is represented by RMSE and scale bars values (mostly below 1 mm). Unlike the results obtained on previous test fields, in this case, it is difficult to identify patterns that would allow to select the best approach.

### 4.6. The Verification of Exterior Orientation Accuracy Performed on Scalebars

The verification of the accuracy of exterior orientation of image sets involved additional control of deviations observed on scale bars located on each wall at each test site as well as on the sensor frame. Reference lengths of the scalebars were measured with a metal ruler with the accuracy of 0.5 mm. In this section, the results obtained for each scenario are presented separately for these two sets of scale bars along with their interpretation.

**Figure 12:**
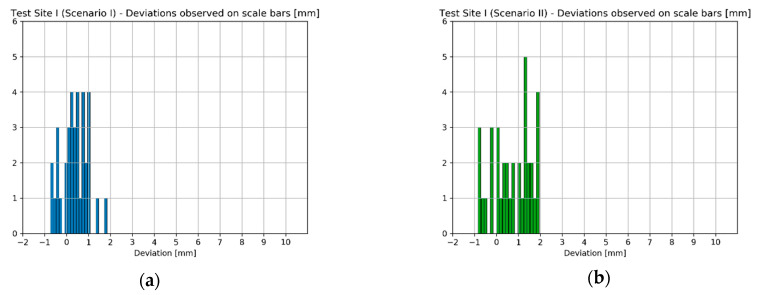

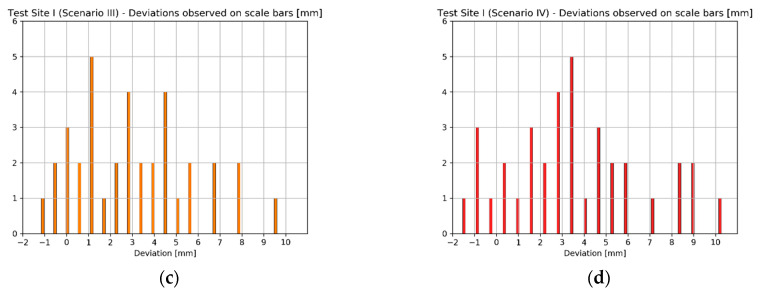
Test Site I: Histograms of deviations observed on scalebars located on the wall (expressed in mm): (**a**) Classical surveying, (**b**) TLS, (**c**) SfM with classical surveying, (**d**) SfM with TLS.

Since the number of EM sensor stations at Test Site I was significantly higher than in case of Test Sites II and III, another way of presenting the results were chosen. The number of observations was enough for histogram generation, so in the case of the Test Site I four histograms illustrating the deviations obtained for each scenario were prepared (Figure 12).

While analysing the histograms of deviations calculated on scalebars with regard to applied orientation method, it can be readily seen that the results acquired in Scenarios I and II as well as Scenario III and IV are pair-wise similar—characterized by similar range of values and deviation distribution. Starting with Scenarios I and II it can be observed that the deviation values range from about −1 mm up to 2 mm. Although the range of values is almost the same in both cases, Scenario I (the “TS” method) should be considered as more accurate since in this case the most observations are within 0–1 mm range, while in case of Scenario II (“TLS” method) the results are more dispersed. Moving on to Scenarios III and IV, the range of deviation values acquired by these two methods is also similar (−1.5 mm up to 10 mm). The accuracy of data registration in case of these two scenarios is approximately equal. It can be concluded that the first two scenarios provided better results—the range of deviation values was much narrower indicating higher accuracy and repeatability of results. In means that in the case of Test Site I the use of SfM (Scenarios III and IV) during data registration achieved worse results. The Test Site I was a plain white wall lacking any characteristic features, which affected the quality of image matching decreasing final accuracy of exterior orientation. Consequently, it can be inferred that for images containing mostly poorly textured areas data registration methods involving the use of SfM for exterior orientation (like Scenario III and IV) are not recommended.

**Figure 13:**
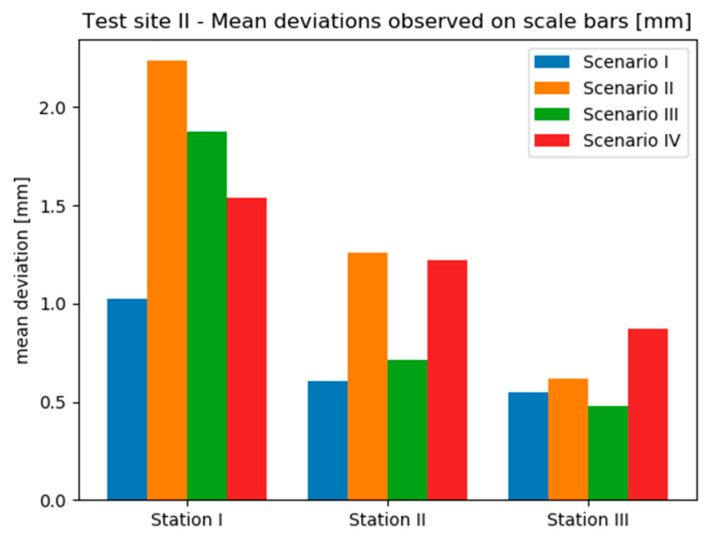
Bar charts illustrating mean deviations observed on scalebars locates on the wall (expressed in mm)—Test Site II.

**Figure 14:**
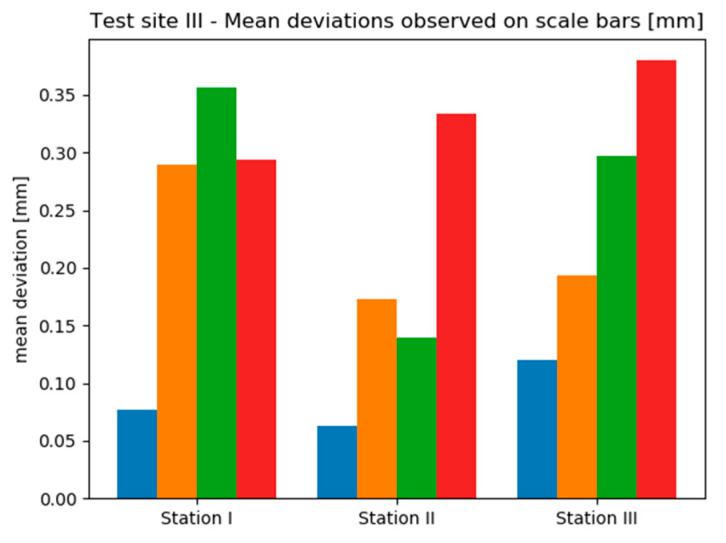
Bar charts illustrating mean deviations observed on scalebars located on the wall (expressed in mm)—Test Site III.

Due to the insufficient amount of data, the deviations occurring on Test Sites II and III were not presented in the form of histograms. Instead, mean deviation values were analysed. Figure 13 and Figure 14 contain bar charts presenting the results.

On the Test Site I mean deviation values range from approximately 0.5 mm up to approximately 2.5 mm. It can be seen that the results obtained by the “TS” method (Scenario I) provided the most similar results on all three stations. Moreover, on two stations this method provided the smallest mean deviations, which do not exceed 1 mm. Only in the case of Station 3, a slightly better effect was achieved by the “TS + SfM” method (Scenario III). Though, it should be noticed that scenarios involving the use of TLS (Scenario II and IV) performed mostly worse than methods relying on geodetic measurements (Scenario I and III). It should be associated with the characteristics of marble, which was the dominant material on the site, and their impact on TLS measurements. Since the TLS technique achieves lower measurement accuracy on translucent materials, in this case the decreased accuracy caused lower accuracy of computation of exterior orientation and thus, higher values of mean deviations observed for Scenarios II and IV.

On the other hand, analysing the results obtained on the Test Site III, it is clearly seen that the observed range of deviations is much narrower than in the case of Test Site II—mean deviations vary approximately between 0.06 mm up to 0.4 mm. On all sensor stations the best results were acquired by the TS method—this time, mean deviations did not exceed 0.15 mm. What is more, comparing the results obtained with and without the use of SfM (so the pairs: Scenario I/III and Scenario II/IV) it can be stated that the use of SfM does not improve the accuracy of exterior orientation. Additionally, in 2 of 3 cases the use of geodetic measurements instead of TLS provided smaller mean deviation values.

To summarized, it might be noticed that the worse results are obtained for Scenarios III and IV. Nevertheless, RMSE values are acceptable and are within the measurement accuracy.

Additional tests involved the calculation of deviations occurring on scalebars determined on the metal frame of the EM sensor. There were 6 signalised reference points installed on the sensor frame and between them three scale bars were defined (Figure 6d).

**Figure 15:**
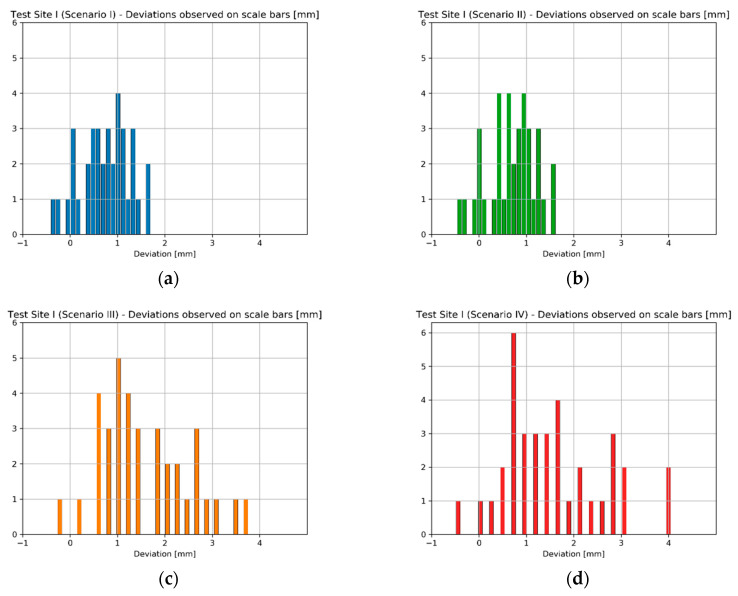
Test Site I: Histograms of deviations observed on scalebars located on the sensor frame (expressed in mm): (**a**) Classical surveying, (**b**) TLS, (**c**) SfM with classical surveying, (**d**) SfM with TLS.

Similar to previous analyses involving scalebars, in this case the deviations were calculated as differences between the reference values and distances computed basing on coordinates derived from the model, according to applied scenario (I–IV). Again, the results obtained on the Test Site I are presented in the form of histograms (Figure 15).

The results obtained on scalebars located on the sensor’s frame again indicate that the accuracy possible to achieve by scenarios I/II and scenarios II/IV is pairwise similar (Figure 15). In the case of TS and TLS methods, the deviation values range from approximately −0.5 mm up to approximately 1.5 mm. The distribution of deviations in both cases is similar. Furthermore, in case of scenarios III and IV the range of deviation values are between approximately −0.5 mm up to approximately 4 mm, being slightly narrower for the Scenario III. This leads to the conclusion that methods of orientation, which do not involve the use of SfM, performed better. It is also worth noting that compared to the results obtained for scalebars located on the wall, the accuracy achieved by Scenario I and II methods is a little higher, but in the case of Scenario III and Scenario IV methods the difference is significant (max deviation on the wall: approximately 10 mm vs max deviation on the sensor frame: approximately 4 mm). Therefore, it can be concluded that SfM-based orientation methods provide better results in case of objects of more complex structure (3D objects)—it is consistent with SfM principles mentioned in previous sections.

**Figure 16:**
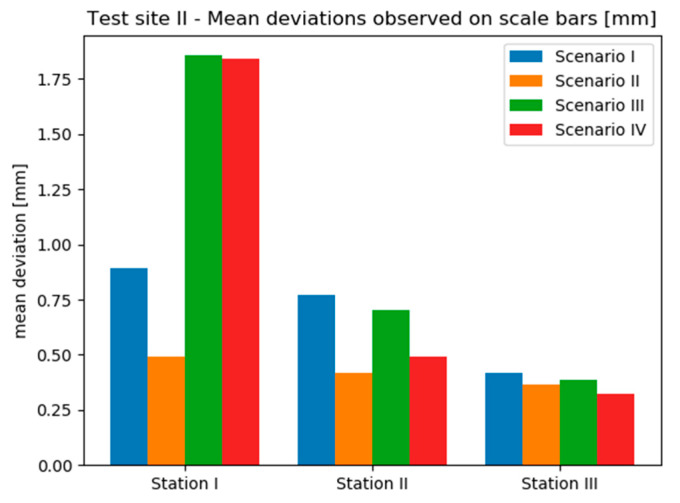
Bar chart illustrating mean deviations observed on scalebars located on the sensor frame (expressed in mm)—Test Site II.

It is readily seen that the results obtained on the Test Site II vary significantly between stations, especially in the case of scenarios III and IV, where mean deviations observed on Station I are several times higher than on the rest of stations (Figure 16). Moreover, on this test site the best results were acquired by the TLS method, which provided both the highest accuracy (mean deviation below 0.5 mm) and repeatability between stations. It should be noted that low accuracy achieved on scale bars located on the wall did not entail the decrease of accuracy on scalebars located on the sensor. However, the differences between scenarios are noticeable only on the first two stations—on the last one the results are comparable and not exceeding 0.5 mm.

**Figure 17:**
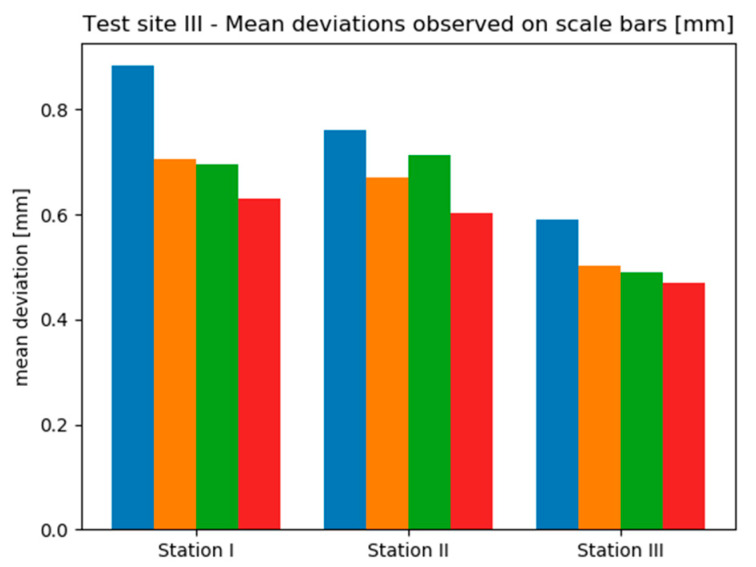
Bar chart illustrating mean deviations observed on scalebars located on the sensor frame (expressed in mm)—Test Site III.

Analysing the results obtained on the Test Site III it can be seen that mean deviations vary in a narrow range between 0.5 mm and 0.9 mm. The differences between scenarios are rather subtle in this case, however, on all stations the Scenario I performed the worst. Nevertheless, basing on Figure 17 it is not possible to determine unequivocally, which method provided the highest accuracy.

Considering deviations calculated on Test Sites II (Figure 16) and III (Figure 17) for scalebars located both on the walls and on the sensor frame, it can be seen that their distributions are contradictory methods, which provided the highest accuracy in the first case and give the worst results in the other.

### 4.7. The Quality Assessment of the Reference Plane Fitting into the Wall + Deformation Analysis

**Figure 18:**
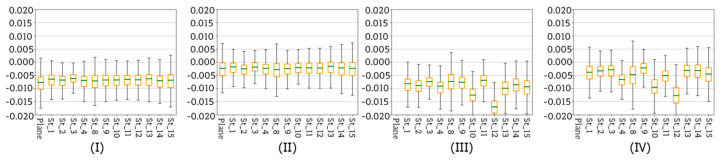
Test Site I: Box plots illustrating the deviations (in meters) of point clouds from the base planes acquired for four scenarios: (**I**)—Classical surveying, (**II**)—TLS, (**III**)—SfM with classical surveying, (**IV**)—SfM with TLS.

**Figure 19:**
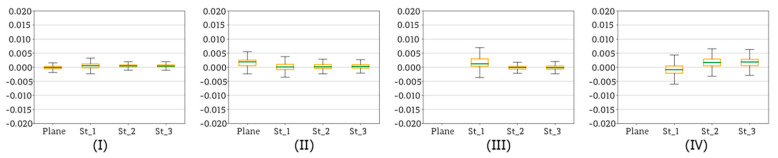
Test Site II: Box plots illustrating the deviations (in meters) of point clouds from the reference planes acquired for four scenarios: (**I**)—Classical surveying, (**II**)—TLS, (**III**)—SfM with classical surveying, (**IV**)—SfM with TLS.

**Figure 20:**
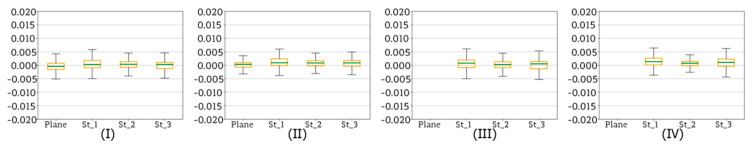
Test Site III: Box plots illustrating the deviations (in meters) of point clouds from the reference planes acquired for four scenarios: (**I**)—Classical surveying, (**II**)—TLS, (**III**)—SfM with classical surveying, (**IV**)—SfM with TLS.

According to the diagram (Figure 5), the next stage of data processing was dense point cloud generation based on terrestrial images. The purpose of this step was to verify the quality of each dense point cloud with regard to corresponding base plane. Dense clouds were generated for image sets covering whole reference walls (as it can be seen in the Figure 6) as well as for the sets of images covering both the sensor and fragments of reference walls, taken at each EM sensor station. Again, the tests considered the results obtained on three test sites, on which four scenarios of data registration were applied. The Figure 18, Figure 19 and Figure 20 illustrate statistics of deviations of cloud points from each base plane (the process base plane definition for each scenario was described in Section 4.1). Each graph presents results obtained for one scenario.

The Test Site I was a plane wall lacking any decorations and thus, characterised by uniform, poor texture. This poor texture visibly affected the accuracy of data registration and the quality of the final dense point cloud. Starting with the analysis of results obtained for scenarios I (Figure 18I) and II (Figure 18II) it is readily seen that the distribution of deviations in both cases is very similar. Nevertheless, in case in Scenario I all values are slightly shifted down indicating the presence of a systematic error which is probably caused by the difficulty in defining proper weights of the observations during the bundle adjustment. The systematic error is approximately 7–8 mm—such values significantly exceed the accuracy of base plane approximation (see the Table 2). In case of Scenario II (Figure 18II) the systematic error can be observed, too, but its value, which ranges between 2 and 3 mm, remains within the measurement accuracy and the accuracy of base plane approximation. Moving on to Scenarios II (Figure 18II) and IV (Figure 18IV), it can be noticed that the proper orientation of data with SfM method on all EM sensor stations was not possible. The range of calculated deviations is fairly wide starting from 3 mm and ending with 17 mm. Observed differences are the consequence of several problems occurring while using SfM and MVS algorithms, like errors during image matching and depth reconstruction which are particularly frequent in case of poorly textured areas. In addition, similar to the Scenario I (Figure 18I), in case of Scenario III (Figure 18III) the presence of a systematic error is visible, too—again its probable cause is decreased quality of bundle adjustment.

Figure 19 and Figure 20 present the results obtained for test sites II and III.

Test Sites II and III were memorial tablets made from marble and sandstone, respectively. Consequently, compared to the Test Site I, both of them were characterized by a more complex texture. Analysing the results obtained for Test Sites II (Figure 19) and III (Figure 20), it can be noticed that deviations of points from the base planes are fairly small and do not exceed 5 mm. This means that compared to the results obtained on the Test Site I, both values of the deviations and their dispersion are smaller, indicating higher accuracy and of data registration and higher repeatability of acquired results. Moreover, in case of both test sites the differences occurring between Scenarios I and II as well as III and IV are small and may be considered as negligible. Such improvement compared to the results obtained on the Test Site I is a consequence of principles of applied SfM and MVS algorithms. Smaller deviation values are the result of fairly complex diverse texture, making it possible to detect more key points of high quality, which resulted in higher accuracy of image matching and higher quality of final dense point cloud.

In conclusion, the choice of a scenario to be applied for data registration, the characteristics of tested object must be taken into consideration. In the case of large areas of plain, uniform texture lacking characteristic features it is recommended to apply methods such as Scenario I and Scenario II, while methods involving SfM are not suitable. On the other hand, all four scenarios are proved to provide similar results in case of objects and areas characterised by diverse texture.

In conclusion, the choice of a scenario to be applied for data registration, the characteristics of tested object must be taken into consideration. In case of large areas of plain, uniform texture lacking characteristic features it is recommended to apply methods such as Scenario I and Scenario II, while methods involving SfM are not suitable. On the other hand, all four scenarios are proved to provide similar results in case of objects and areas characterised by diverse texture.

### 4.8. The Quality Assessment of the Planes Fitting into the Multi-View Stereo (MVS) Point Clouds/Surveying

**Figure 21:**
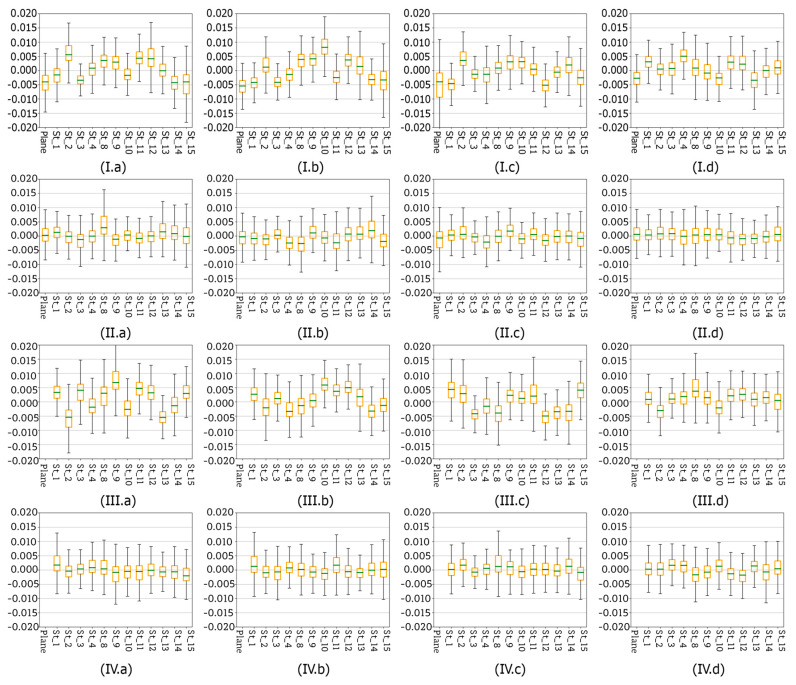
Test Site I: Box plots illustrating the deviations (in meters) of point clouds subsampled with different sampling radius from the reference planes fitted into Multi-View Stereo (MVS) point clouds: (**a**) 5 mm, (**b**) 1 cm, (**c**) 3 cm, (**d**) 5 cm for four scenarios (I—Classical surveying, II—TLS, III—SfM with classical surveying, IV—SfM with TLS).

**Figure 22:**
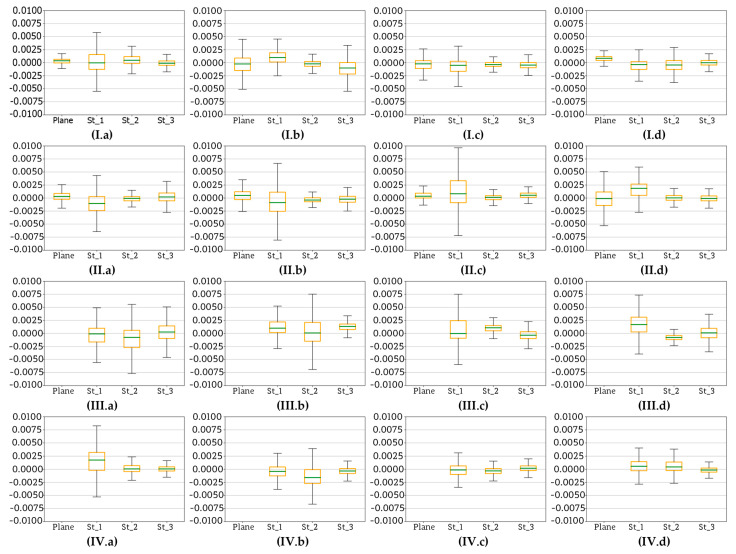
Test Site II: Box plots illustrating the deviations (in meters) of point clouds subsampled with different sampling radius from the reference planes fitted into MVS point clouds (**a**) 5 mm, (**b**) 1 cm, (**c**) 3 cm, (**d**) 5 cm for four scenarios (I—Classical surveying, II—TLS, III—SfM with classical surveying, IV—SfM with TLS).

**Figure 23:**
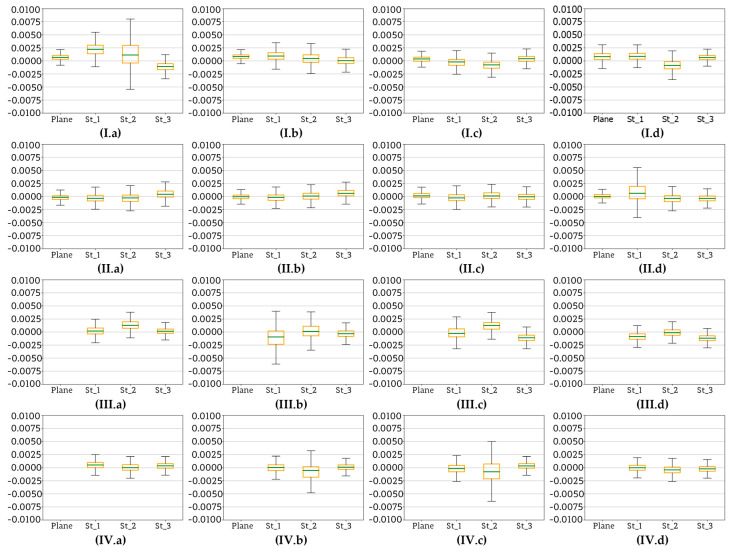
Test Site III: Box plots illustrating the deviations (in meters) of point clouds subsampled with different sampling radius from the reference planes fitted into MVS point clouds (**a**) 5 mm, (**b**) 1 cm, (**c**) 3 cm, (**d**) 5 cm for four scenarios (I—Classical surveying, II—TLS, III—SfM with classical surveying, IV—SfM with TLS).

In the previous Section 4.7 reference planes (base planes) were determined by fitting them into two sets of source data: control points measured with a total station and control points measured with a TLS. However, the authors decided to check if it is possible to avoid the use of additional measuring devices (like a total station or TLS) at this stage. In order to verify this hypothesis, a set of reference planes were fitted into MVS point clouds which were then subsampled with different resolution. Authors decided to use the subsampling method implemented in the Open3D library [86]. For this purpose, four subsampling radius values were chosen: 5 mm, 1 cm, 3 cm, and 5 cm. Point clouds subsampled with different resolution were compared to four reference planes—each one fitted into an MVS point cloud generated for each scenario. Figure 21, Figure 22 and Figure 23 show the deviations calculated for point clouds acquired in all four scenarios. Again, the deviations are presented in the form of box plots showing basic statistics.

Analysing sets of box plots (Figure 21) prepared for each scenario (I–IV), at first glance some trend can be observed. There are similarities which can be observed in case of a pair: Scenario I and Scenario III as well as for the pair Scenario III and Scenario IV. Consequently, the results obtained with these pairs of methods will be analysed separately. Starting with the Scenarios I and III it can be observed that mean deviation values do oscillate around zero, but the range of obtained values is quite wide in both cases, visibly wider that in the case of Scenarios II and IV. Moreover, especially in the case of Scenario III it can be seen that the accuracy improves with decreasing resolution of the MVS reference point cloud, and it can be concluded that lower resolution allows the elimination of some errors present in the original data. Mean values of deviations calculated for Scenarios II and IV are close to zero. Additionally, the box plot sizes and both minimum and maximum values are similar. Like in Scenarios I and III, in the second scenario a slight improvement of accuracy achieved for the point with 5 cm resolution was observed. On the other hand, such a phenomenon was not the case in Scenario IV. Furthermore, it should be stressed that in all cases the maximum deviations do not exceed +/−1.5 cm.

For the Test Site II (Figure 22) results shows that the deviations between the dense point cloud and plane fitted into the downsampled point cloud do not exceed +/−2 mm (yellow boxplot). The best results were obtained for Scenario I. Similar to the previous results, it might be seen that SfM method (Scenarios III and IV) allows to obtain worse results and the size of the boxplot is higher. In the case of the Test Site III (Figure 23), similar to the Test Site II, better results were obtained in the case of the Scenario I and also for Scenario II. The worst results were obtained by the Scenarios III and IV, but they are still acceptable.

### 4.9. The Analysis of Skews and the Orientation Angled of the Sensor Frame

The final stage of the evaluation of methods for exterior orientation determination involved the examination of two additional factors—the influence of the reference plane determination with regard to the base plane and the quality of assessment of the sensor orientation in relation to the base plane. The process of base plane definition corresponding to walls and the calculation of reference planes for each sensor station were described in detail in Section 4.1 and Section 4.7. The aim of the analyses presented below is to examine the spatial relations between the base planes determined for each test site and scenario and the reference planes calculated using dense point clouds generated for each sensor station. For each test site and orientation scenario (I–IV) the skews between mentioned pairs of planes were computed. Table 6 contains mean linear values of skews (Equation (16)) divided into X, Y, and Z components for reference planes fitted into marked points in relation to the base planes.

In order to analyse the linear deviation, the skew values were changed into the linear values in the following form:

(16) $$\begin{array}{l} X=16\;\left[cm\right]*\sin\left(\omega \right)\\ Y=16\;\left[cm\right]*\sin\left(\varphi \right)\\ Z=11\;\left[cm\right]*\sin\left(\kappa \right)\;, \end{array}$$

where 11 cm is the half of the width of the frame, 16 cm is a half of the height and depth of the frame, ω,φ,κ is the skew angels of the plane.

Table 7 presents the mean values of skews calculated for reference planes fitted into dense point clouds resampled with 3 cm resolution in relation to the base planes.

Analysing the results obtained for the Test Site I, no significant differences were observed either with regard to the way the reference plane was defined, or the applied orientation scenario (Table 6 and Table 7). All skew values are similar and not exceeding the measurement accuracy. On the contrary, the results acquired on test sites II and III are much more varied depending on the scenario. It is a consequence of higher values of measurement errors appeared both in case of geodetic surveys and the TLS measurements. Nevertheless, the skew values are within the measurement accuracy.

The last stage of data processing is the computation of the orientation angles of the sensor: ω,φ,κ. To calculate these angles, the rigid-body orientation approach described in the Section 2.5 was applied. Next, the distances between the vertexes of the sensor frame and the wall were measure with the use of a metal ruler, that served as a reference. Based on these distances, the angles ω,φ,κ were calculated once more, providing the second set of data for comparison. The analysis of differences occurring between these two sets of angles was performed for all three test sites and all four orientation scenarios. The results are presented in Table 8 and Table 9. The angular values were then converted into linear values (see Equation (16)). In the first case (Table 8) the base plane determined using marked control points served as a reference plane. In the second case (Table 9), the reference plane fitted into a reference dense point cloud resampled with 3 cm resolution, generated for each sensor station, was used as a reference.

The results presented in Table 8 and Table 9 contain final accuracy of the sensor orientation angles achieved with the use of four orientation methods. First, it can be seen that there are no significant differences in acquired results, depending on the choice of the reference plane, as their values range between 0–1.2 mm. The accuracy achieved for Test Sites II and III is correct and considering the absolute deviation values ranging from 0 mm up to 3.9 mm, they are within the measurement accuracy of classic measurements. However, the deviations observed on the Test Site I are slightly higher than the others (above 4 mm) despite the use of more accurate measurement methodology. The probable cause of this phenomenon can be a small systematic error.

## 5. Conclusions

The preparation of comprehensive architectural documentation of a monumental object is one of the key factors in the field of preservation of cultural heritage. The value of such documentation increases with the number of data sources and measuring techniques used for its creation. The electromagnetic sensor, which is a component of the proposed multisensor platform for structural health monitoring, makes it possible to examine the state of conservation of a cultural heritage object together as well as to investigate its inner structure and destructive processes (moisture, cracks) causing its progressing degradation. The integration of measurements performed with this sensor and with data acquired by other measurement techniques (e.g., terrestrial laser scanning, close-range photogrammetry) would extend the scope of possible structural analyses with regard to the “spatial element”—the spatial distribution of occurring phenomena and the object features. However, for this purpose, the multi-source data needs to be georeferenced in the same referenced system. Until now, there had been no methodology proposed for the determination of the EM sensor system’s geolocalisation. Furthermore, the orientation of the sensor in relation to the investigated object directly affects the sensor response. Consequently, known orientation parameters are necessary for the calculation of corrections which need to be applied to the observations in order to make them correct and comparable in case of differential multitemporal analyses. In this article the Authors proposed a methodology for geolocalising the EM sensor in an assumed external reference system and to determine the orientation parameters with the accuracy characterized by RMSE not exceeding 5 mm. In order to find the parameters of relative and exterior orientation, three measuring techniques were applied: classical surveying, terrestrial laser scanning and close-range photogrammetry—Structure-from-Motion and MultiView-Stereo.

The whole experimentation consisted on three main steps: (1) data acquisition and pre-processing (including the extraction of the reference plane and the determination of coordinates of reference points); (2) sensor orientation and bundle adjustment, and (3) the estimation of exterior and relative orientation parameters together with accuracy analyses and the quality assessment.

The study of orientation methods possible to be applied for the sensor involved four different approaches (scenarios). In order to check which of the proposed scenarios would provide the highest accuracy of resulting relative and exterior orientation parameters, a series of analyses were performed. These analyses covered the following issues: (1) the accuracy analysis concerning the determination of the base plane (which was used later during relative orientation computation and at further stages of data processing) based on two sources of data: total station measurements and TLS data, (2) the accuracy analysis of the relative orientation of the images covering the walls and the sensor at each station, based on the analysis of reprojection error values, (3) the accuracy analysis of exterior orientation parameters using RMSE values obtained on control and check points, additional control using scale bars located on the walls and on the sensor’s frame, (4) the analyses concerning the accuracy of reference plane approximation based on MVS dense point clouds and the accuracy of the orientation of the sensor in relation to mentioned reference plane. Consequently, the accuracy of relative and exterior orientation of the EM sensor should be considered separately.

The reprojection error is an indicator of the quality of relative orientation of images and therefore, it allows to verify the correctness of a photogrammetric model. A set of properly aligned photos should be characterized by the reprojection error, which value does not exceed considerably 1 pixel, regardless of the quality of the object texture. However, significant differences are to be observed in the maximum reprojection error values. 

The inclusion of control points during the bundle adjustment should not negatively affect the final accuracy of the process. In fact, using them as manually identified tie points should rather increase the accuracy of relative orientation. Differences observed in minimum, maximum, and mean reprojection error values are dependent of the effectiveness of feature detection algorithms and they are particularly visible in case of poorly textures areas containing a small number of features. Additional, manually determined tie points should considerably improve the accuracy of relative orientation of images characterized by uniform texture and minimise the reprojection error. Nevertheless, in case of photographs of complex, diverse texture the addition of extra tie points would not result in a significant improvement in bundle adjustment accuracy. However, it is noteworthy that the specification of proper observation weights is crucial as wrong weights may result in the increase of the reprojection error value or even make it impossible to calculate the relative orientation parameters.

Based on the quality assessment of the relative orientation performed on image sets covering only the reference walls, it can be concluded that on all three test sites proper weights were assigned to the observations and the relative orientation was calculated properly (Figure 7). In case of the Test Site I, which was a plain wall of poor, uniform texture, the addition of control points measured by TLS (Scenario II) as manual tie points improved the accuracy of bundle adjustment decreasing the mean reprojection error twice. However, in case of test sites II and III, which were characterized with more complex texture rich of features, the addition of TLS control points did not influence the final relative orientation accuracy. Moreover, the use of TS (Scenario I) control points did not influence the accuracy of bundle adjustment in the case of test sites I and III and what is more, in the case of Test Site II, mean reprojection error increased. 

Moving on to the relative orientation of images covering both the EM sensor the reference walls, it is clear that the relative orientation of the image sets obtained on all test sites was calculated properly in all scenarios as the mean reprojection error only in a few cases slightly exceeded 1 pixel, mostly remaining below 1 pixel. On the grounds of performed analyses it can be concluded that at the stage of relative orientation computation the choice of orientation scenario is not a factor, which affects the accuracy of bundle adjustment significantly.

In order to calculate the exterior orientation of images as well as to assess the accuracy of performed computation, two types of reference points of known coordinates were used. Control points were included in the adjustment process. Check points were used to check the accuracy of resulting photogrammetric model. RMSE values obtained on check points are the independent verification of the model accuracy since their coordinates in an external coordinate system are computed based on exterior orientation parameters and their location on images expressed in pixels. Then, their coordinates derived from the photogrammetric model are compared with reference coordinates measured with another technique (in this case: classical surveying and TLS). To avoid the influence of errors coming from the transformation between the local coordinate system of the scanner and the external coordinate system of each test site, the Authors decided to examine the linear values of RMSE obtained on control and check points since they are independent on the applied reference system. Additionally, the accuracy of exterior orientation was verified using scalebars established both on the reference walls and on the sensor’s frame. Their lengths were measured with the accuracy of 0.5 mm with a metal ruler. 

The analysis performed on reference points suggests that compared to TLS, classical surveying allowed to achieve higher accuracy of exterior orientation on all three test sites, especially in case of Test Site II, where the difference between the results acquired with these two methods is particularly visible (Figure 8). However, it must be stressed that RMSE values obtained for TS do not exceed 0.5 mm and in case of TLS measurements the max RMSE is 1.2 mm—both results indicate high accuracy of exterior orientation of the images and are acceptable. Moving on to the analysis concerning the accuracy of exterior orientation of the images covering both the EM sensor and the reference walls, it can be concluded that we managed to achieve high orientation accuracy on all test sites as the RMSE values are mostly below 3 mm on each sensor station. Furthermore, the differences between RMSE values obtained for Scenario I and II are less than 2 mm. Analysing the deviations observed on scalebars on the Test Site I (Figure 12 and Figure 15), it was noticed that the results obtained for scenarios I/II and II/IV are pairwise similar, indicating that scenarios I and II provide higher accuracy than the methods involving the use of SfM. The probable reason is the plain texture of this test site. The differences between all four scenarios are very subtle on remaining test sites (Figure 13, Figure 14, Figure 16 and Figure 17) and can be considered as negligible. Moreover, comparing the deviations obtained on scalebars located on the wall and these calculated for the sensor’s frame, it can be concluded that SfM-based orientation methods provide better results in case of characterized by more complex structure (3D objects).

The positions of the sensor and the examined monumental object relative to each other need to be known before the sensor data are processed. To determine the relative position of the sensor, the reference plane needs to be established. For this purpose, two different approaches were applied in order to determine the parameters of abovementioned reference plane (the “base plane”). The first approach relied on the determination of the base plane parameters using the control points, which coordinates were measured by two techniques: classical surveying and TLS (Table 1). The second approach used dense point clouds resulting from further processing of the images with the use of SfM/MVS algorithms (see the Section 4.8 and Section 4.9). The Authors decided to analyse the results obtained by these two approaches separately, because the first one relies on point coordinates measured directly while in the second case the plane was fitted into a dense cloud which is a result of several subsequent stages of processing of an image set. The analyses performed lead to the conclusion that the use of signalised control points provided higher accuracy of determination of the base plane parameters, regardless on the used scenario (I–IV). The measurement accuracy is also influenced by the way the centre of the marked point is determined. On the Test Site I the points were measured from three stations and their coordinates were calculated in the process of geodetic adjustment. On the contrary, on test sites II and III only one instrument station was established, and the points were measured with the polar method. In case of the TLS measurements, the centre of the marker was determined by means of statistical template matching. The use of automatic detection algorithm based on statistical analysis made it possible to eliminate the observer errors, which are a common cause of blunders occurring in classical surveying. In addition, it is worth noting that the amount of time needed to determine the point coordinates with the use of automatic marker detection performed on TLS data is approximately three times shorter than measurements performed with a total station. It is a very important advantage in cases where a cultural heritage objects is concerned. In cases concerning monumental objects, the possible time of measurement is limited due to the tourist traffic and conservation works.

The next series of analyses concerned the quality assessment of reference plane determination based on different data sources, regarding the base planes. The first type of reference planes was determined, using the marked points—the same point sets as those used in the base plane determination, but with coordinates calculated in the bundle adjustment. The second type of reference planes was created for each scenario by fitting planes into dense point clouds resampled with the resolution of 3 cm. For each type, the linear deviations from base planes were calculated (this step was described in detail in the Section 4.9). Comparing the results obtained by TLS and classical surveying, it can be observed that higher accuracy was achieved in case of TLS measurements—only on the Test Site I the results obtained with these two methods were comparable (Table 6). However, it should be noticed that on this test site the accuracy was improved thanks to the increased number of measurement stations.

Before using TLS point clouds as source data for reference plane determination, the material from which the examined object is made must be taken into consideration. It has been proved that the accuracy of TLS measurements is low in case of objects made from highly reflective and translucent materials, such as marble, which is particularly common in historical objects. For this reason, in case of such objects TLS is not recommended as source data. 

In the case of the Test Site II, where marble was a dominant material, the shape deformations (the deviations from the base planes) reached 1.7 cm. Comparing the results obtained for MVS dense point clouds and for signalised points, it can be seen on Test Sites II and III the approach using signalised points higher accuracy, for the Scenarios II and IV and in the case of the Test Site I similar for all Scenarios (Table 7). Despite the observed differences, the deviations obtained are within the measurement accuracy. The analysis of skews (Table 8) requires the results obtained on Test Site I to be discussed separately from test sites II and III since the structure of the object on this site as well as its texture varies significantly from the others. As mentioned before, unlike test sites II and III, which are characterised by complex and diverse texture, Test Site I is a plain white wall lacking any decorations or other characteristic features. Analysing the deviation values, it can be observed that the deviations obtained on Test Site I are higher than these calculated on test sites II and III. However, the maximum difference between the results achieved on these test sites hardly exceeds 3 mm, remaining below 2 mm in most cases. This means that these differences are within the measurement accuracy and can be considered as negligible. The analysis of results obtained for each sensor station (Table 9) leads to similar conclusions.

On the grounds of the research, it can be concluded that it is impossible to propose one versatile workflow for determination of exterior orientation parameters of the electromagnetic sensor and its orientation in relation to an examined object. All proposed scenarios for relative orientation of the sensor allow to calculate necessary corrections, which need to be applied to the signal sent and received by the sensor due to the distance and tilt between the sensor and the object. The investigation of exterior orientation methods revealed several aspects, which need to be taken into account. Firstly, in cases when the possible time of measurement is strictly limited, terrestrial laser scanning is a fast technique providing sufficient accuracy of coordinates of points forming geodetic network. However, it is impossible to establish the measurement station in the same position again in the next measurement session in order to perform multitemporal analysis. For this purpose, the use of a total station is required. 

The choice of method, which should be applied in order to determine the reference plane (the “base plane”), is strictly dependent on the characteristics of the examined object. In the case of objects of simple structure and plain texture it is recommended to either use the TLS technique or, if possible, marked control points. In the case of such objects it is not recommended to apply methods involving the SfM algorithms since a small number of features results in decreased accuracy of the final adjustment. For this reason, SfM-based approaches like Scenarios III and IV are not suitable for the determination of the sensor’s position in the case of such objects. Furthermore, objects of non-highly reflective and non-transparent surface characterised by diverse texture (like in the case of Test Site III) allows the application of TLS measurements, marked control points, and MVS dense point clouds to determine the parameters of a base plane. However, TLS-based methods are not recommended for marble surfaces, which are very common in cultural heritage objects. In case of marble and other translucent or semi-translucent structures, it is possible to determine the sensor positions by applying methods based on signalised control points (Scenario I and II) and if the marked points cannot be placed, then the SfM-based methods (Scenario III and IV) can be used.

## Figures and Tables

**Table 1:** RMSE values of the reference points coordinates—the table includes the results obtained with both measuring methods considering all stations of the instrument.

Test Site	Surveying			Terrestrial Laser Scanning
	RMSE [mm]			
	X	Y	Z	Linear
I	0.2	0.3	0.1	2.3
II	2.0	2.0	2.0	2.2
III	2.0	2.0	2.0	1.8

**Table 2:** Quality assessment of the reference plane determination.

Test Site	No. of Points		Surveying		Terrestrial Laser Scanning		
	Control	Check	Linear Deviations from the Plane [mm]				
			Control	Check	Marked Control	Marked Check	Natural Check
I	3	4	0.6	4.1	0.4	1.6	3.8
II	3	0	0.1	X	0.2	X	17.5
III	3	2	0.1	1.3	0.9	0.9	3.6

**Table 3:** Relative orientation of the chosen EM sensor stations [in pixels]—Test site I.

Scenario		Station 1			Station 2			Station 3		
		Min	Max	Mean	Min	Max	Mean	Min	Max	Mean
SFM	Wall	0.1	2.7	0.6	0.0	2.7	0.8	0.1	1.5	0.8
	Sensor	0.0	1.9	0.5	0.0	1.6	0.5	0.1	1.2	0.5
I	Wall	0.1	2.7	0.6	0.0	2.7	0.8	0.1	1.5	0.8
	Sensor	0.0	1.9	0.5	0.0	1.6	0.5	0.1	1.2	0.6
II	Wall	0.1	2.7	0.6	0.0	2.7	0.8	0.1	1.5	0.8
	Sensor	0.0	1.9	0.5	0.0	1.6	0.5	0.1	1.2	0.5
III	Wall	0.1	2.7	0.6	0.0	2.7	0.8	0.1	1.5	0.8
	Sensor	0.0	1.9	0.5	0.0	1.6	0.5	0.1	1.2	0.5
IV	Wall	0.1	2.7	0.6	0.0	2.7	0.8	0.1	1.5	0.8
	Sensor	0.0	1.9	0.5	0.0	1.6	0.5	0.1	1.2	0.5

**Table 4:** Relative orientation of the EM sensor [in pixels]—Test site II.

Scenario		Station 1			Station 2			Station 3		
		Min	Max	Mean	Min	Max	Mean	Min	Max	Mean
SFM	Wall	0.1	0.6	0.4	0.0	1.2	0.5	0.0	1.5	0.6
	Sensor	0.0	0.8	0.4	0.0	0.8	0.5	0.0	1.4	0.5
I	Wall	0.1	1.3	0.9	0.1	1.9	0.7	0.0	2.0	0.7
	Sensor	0.0	1.2	0.6	0.0	1.1	0.7	0.1	1.8	0.8
II	Wall	0.1	0.6	0.4	0.0	1.2	0.5	0.0	1.5	0.6
	Sensor	0.0	0.8	0.4	0.0	0.8	0.5	0.0	1.4	0.5
III	Wall	0.1	0.6	0.4	0.0	1.2	0.5	0.0	1.5	0.6
	Sensor	0.0	0.8	0.4	0.0	0.8	0.5	0.0	1.4	0.5
IV	Wall	0.1	0.6	0.4	0.0	1.2	0.5	0.0	1.5	0.6
	Sensor	0.0	0.8	0.4	0.0	0.8	0.5	0.0	1.4	0.5

**Table 5:** Relative orientation of the EM sensor [in pixels]—Test site III.

Scenario		Station 1			Station 2			Station 3		
		Min	Max	Mean	Min	Max	Mean	Min	Max	Mean
SFM	Wall	0.1	2.1	0.8	0.0	2.1	0.9	0.0	1.2	0.5
	Sensor	0.1	1.7	0.9	0.2	2.3	1.3	0.1	3.3	1.2
I	Wall	0.1	1.2	0.6	0.1	1.1	0.5	0.0	0.8	0.4
	Sensor	0.1	1.3	0.7	0.1	1.6	1.0	0.1	1.1	0.7
II	Wall	0.1	1.1	0.6	0.0	1.2	0.5	0.0	0.8	0.4
	Sensor	0.1	2.3	1.0	0.1	2.5	1.3	0.1	3.4	1.3
III	Wall	0.1	2.1	0.8	0.0	2.1	0.9	0.0	1.2	0.5
	Sensor	0.1	1.7	0.9	0.2	2.3	1.3	0.1	3.3	1.2
IV	Wall	0.1	2.1	0,8	0.0	2.1	0.9	0.0	1.2	0.5
	Sensor	0.1	1.7	0.9	0.2	2.3	1.3	0.1	3.3	1.2

**Table 6:** Mean values of linear skews between the planes created using signalized points and base planes.

Scenario	Test Site 1			Test Site 2			Test Site 3		
	dX [mm]	dY [mm]	dZ [mm]	dX [mm]	dY [mm]	dZ [mm]	dX [mm]	dY [mm]	dZ [mm]
I	0.28	0.54	0.26	3.46	1.77	0.22	3.50	1.78	0.03
II	0.15	0.93	0.18	0.19	0.08	0.18	0.70	2.68	0.10
III	0.53	1.04	0.27	3.63	1.86	0.21	3.00	1.53	0.10
IV	0.22	1.36	0.40	0.68	0.28	0.54	0.63	2.40	0.09

**Table 7:** Mean values of linear skews between the planes formed from point clouds (resampling 3 cm) and base planes.

Scenario	Test Site 1			Test Site 2			Test Site 3		
	dX [mm]	dY [mm]	dZ [mm]	dX [mm]	dX [mm]	dY [mm]	dZ [mm]	dY [mm]	dX [mm]
I	0.59	1.16	0.72	3.56	1.82	0.05	3.52	1.80	1.47
II	0.20	1.25	0.59	1.35	0.53	1.02	1.35	5.11	1.02
III	0.46	0.91	0.42	0.56	0.28	0.43	1.84	0.93	1.01
IV	0.17	1.10	0.87	0.62	0.24	0.56	1.72	6.55	0.71

**Table 8:** Mean skew deviation of the EM sensor (relative to the base plane).

Scenario	Test Site 1			Test Site 2			Test Site 3		
	dX [mm]	dY [mm]	dZ [mm]	dX [mm]	dY [mm]	dZ [mm]	dX [mm]	dY [mm]	dZ [mm]
I	4.4	0.3	0.7	2.4	1.3	0.0	2.6	1.2	−2.0
II	4.3	1.5	0.7	1.1	1.8	−0.2	3.7	3.4	−2.1
III	4.4	0.7	0.9	1.1	1.8	0.3	2.6	1.2	−2.1
IV	4.3	1.4	0.9	1.1	1.8	−0.1	3.7	3.4	−2.2

**Table 9:** Mean skew deviations of the EM sensor (relative to the reference plane fitted into resampled dense point cloud).

Scenario	Test Site 1			Test Site 2			Test Site 3		
	dX [mm]	dY [mm]	dZ [mm]	dX [mm]	dY [mm]	dZ [mm]	dX [mm]	dY [mm]	dZ [mm]
I	4.1	1.1	1.8	3.2	0.8	0.0	3.5	0.6	−1.3
II	4.2	2.2	1.9	0.9	1.8	0.5	3.9	2.5	−1.8
III	4.2	1.0	1.8	2.6	1.2	0.2	3.5	0.6	−1.6
IV	4.3	2.0	1.8	1.3	1.6	0.2	3.5	4.2	−1.9

## References

[1] ECHOES Heritage Conservation & Regeneration. https://www.echc.eu/wp-content/uploads/2019/04/Position-Paper-FP9_RM_revised_low.pdf.

[2] Stylianidis E. (2019). CIPA-Heritage Documentation: 50 Years: Looking Backwards. ISPRS Int. Arch. Photogramm. Remote Sens. Spat. Inf. Sci..

[3] Gil Z. Obiekty Zabytkowe Bez Rys. https://www.renowacjeizabytki.pl/artykuly-techniczne/Obiekty-zabytkowe-bez-rys,1408.

[4] ICOMOS/Iscarsah Committee, Recommendations for the Analysis, Conservation and Structural Restoration of Architectural Heritage. https://www.icomos.org/en2003.

[5] COMOS *CHARTER,* Principles for the Analysis, Conservation and Structural Restoration of Architectural Heritage. http://www.icomos.org/charters/structures_e.pdf.

[6] Lourenço P.B. (2013). Conservation of cultural heritage buildings: Methodology and application to case studies. Rev. Alconpat.

[7] Bláha J., Novotný J. (2018). Report Assessing Innovative Restoration Techniques, Technologies and Materials Used in Conservation.

[8] Bruno S., Fatiguso F. (2018). Building conditions assessment of built heritage in historic building information modeling. Int. J. Sustain. Dev. Plan..

[9] Gattulli V., Lepidi M., Potenza F. (2016). Dynamic testing and health monitoring of historic and modern civil structures in Italy. Struct. Monit. Maint..

[10] Kot P., Shaw A., Jones K.O., Cullen J.D., Mason A., Al-Shamma’a A.I. (2014). The feasibility of using electromagnetic waves in determining the moisture content of building fabrics and the cause of the water ingress. Proc. Int. Conf. Sens. Technol. ICST.

[11] Gkantou M., Muradov M., Kamaris G.S., Hashim K., Atherton W., Kot P. (2019). Novel Electromagnetic Sensors Embedded in Reinforced Concrete Beams for Crack Detection. Sensors.

[12] Teng K.H., Kot P., Muradov M., Shaw A., Hashim K., Gkantou M., Al-Shamma’a A. (2019). Embedded smart antenna for non-destructive testing and evaluation (NDT&E) of moisture content and deterioration in concrete. Sensors.

[13] Kot P., Shaw A., Riley M., Ali A.S., Cotgrave A. (2017). The Feasibility of Using Electromagnetic Waves in Determining Membrane Failure Through Concrete. Int. J. Civ. Eng..

[14] Kot P., Ali A.S., Shaw A., Riley M., Alias A. (2016). The application of electromagnetic waves in monitoring water infiltration on concrete flat roof: The case of Malaysia. Constr. Build. Mater..

[15] Tobiasz A., Markiewicz J.S., Lapinski S., Nikel J., Kot P., Muradov M. (2019). Review of Methods for Documentation, Management and Sustainability of Cultural Heritage. Case Study: Museum of King Jan III ’ s Palace at Wilanów. Sustainability.

[16] Puertolas Montañez J.A., Mendoza Rodriguez A., Sanz-Prieto I. (2013). Smart Indoor Positioning/Location and Navigation: A Lightweight Approach. Int. J. Artif. Intell. Interact. Multimed..

[17] Mautz R. (2012). Indoor Positioning Technologies.

[18] Sakpere W., Adeyeye Oshin M., Mlitwa N.B.W. (2017). A state-of-the-srt survey of indoor positioning and navigation systems and technologies. S. Afr. Comput. J..

[19] Amsters R., Stevens N., Lauwers Q. Evaluation of Low-Cost / High-Accuracy Indoor Positioning Systems. Proceedings of the Fourth International Conference on Advances in Sensors, Actuators, Metering and Sensing-ALLSENSORS 2019.

[20] Jekabsons G., Kairish V., Zuravlyov V. (2011). An Analysis of Wi-Fi Based Indoor Positioning Accuracy. Sci. J. Riga Tech. Univ. Comput. Sci. Appl. Comput. Syst..

[21] Mahfouz M.R., Zhang C., Merkl B.C., Kuhn M.J., Fathy A.E. (2008). Investigation of High-Accuracy Indoor 3-D Positioning Using UWB Technology. IEEE Trans. Microw. Theory Tech..

[22] Mraz L. Accuracy Considerations for UWB Indoor Tracking in an Industrial Environment. https://www.sewio.net/accuracy-considerations-for-uwb-indoor-tracking-in-an-industrial-environment/.

[23] Zafari F., Gkelias A., Leung K.K. (2019). A Survey of Indoor Localization Systems and Technologies. IEEE Commun. Surv. Tutor..

[24] Woźniak M., Odziemczyk W., Nagórski K. (2013). Investigation of practical and theoretical accuracy of wireless indoor positioning system Ubisense. Rep. Geod. Goeinform..

[25] Marcellino D. An Examination of Ultra-Wideband (UWB) for Positioning & Location Tracking. https://www.airfinder.com/blog/ultra-wideband-positioning-location-tracking.

[26] Xu H., Ding Y., Li P., Wang R., Li Y. (2017). An RFID Indoor Positioning Algorithm Based on Bayesian Probability and K-Nearest Neighbor. Sensors.

[27] Wang J., Adib F., Knepper R., Katabi D., Rus D. (2013). RF-Compass: Robot Object Manipulation Using RFIDs. Proceedings of the 19th Annual International Conference on Mobile Computing & Networking-MobiCom’13.

[28] Shen L., Zhang Q., Pang J., Xu H., Li P., Xue D. (2019). ANTspin: Efficient Absolute Localization Method of RFID Tags via Spinning Antenna. Sensors.

[29] Davidson P., Piché R. (2017). A Survey of Selected Indoor Positioning Methods for Smartphones. IEEE Commun. Surv. Tutor..

[30] Bonde G.D., Pal S.R., Barwal P.U., Khan S.I., Ablankar K. (2015). Finding Indoor Position of Person Using Wi-Fi & Smartphone: A Survey. Int. J. Innov. Res. Sci. Technol..

[31] Banerjee S., Suski W., Hoover A. Sensor Set Switching Noise in UWB Indoor Position Tracking. Proceedings of the IEEE International Conference on Ultra-Wideband.

[32] Hazas M., Hopper A. (2006). Broadband Ultrasonic Location Systems for Improved Indoor Positioning. IEEE Trans. Mob. Comput..

[33] Li J., Han G., Zhu C., Sun G. (2016). An Indoor Ultrasonic Positioning System Based on TOA for Internet of Things. Mob. Inf. Syst..

[34] Ijaz F., Yang H.K., Ahmad A.W., Lee C. Indoor Positioning: A Review of Indoor Ultrasonic Positioning systems. Proceedings of the the 15th International Conference on Advanced Communications Technology—ICACT2013.

[35] Mautz R., Tilch S. Survey of optical indoor positioning systems. Proceedings of the International Conference on Indoor Positioning and Indoor Navigation.

[36] Mendoza-Silva G.M., Torres-Sospedra J., Huerta J. (2019). A Meta-Review of Indoor Positioning Systems. Sensors.

[37] Faragher R.M., Sarno C., Newman M. Opportunistic radio SLAM for indoor navigation using smartphone sensors. Proceedings of the 2012 IEEE/ION Position, Location and Navigation Symposium.

[38] Kahmen H., Faig W. (2012). Surveying.

[39] Teunissen P.J.G. (2003). Adjustment Theory.

[40] Börlin N., Murtiyoso A., Grussenmeyer P., Menna F., Nocerino E. (2018). Modular bundle adjustment for photogrammetric computations. ISPRS Int. Arch. Photogramm. Remote Sens. Spat. Inf. Sci..

[41] Kraus K. (2007). Photogrammetry, Geometry from Images and Laser Scans, Second Edition, Karl Kraus.pdf.

[42] Berenyi A., Lovas T., Barsi A. Terrestrial Laser Scanning–Civil Engineering Applications. Proceedings of the International Archives of Photogrammetry Remote Sensing and Spatial Information Sciences.

[43] Liang X., Kankare V., Hyyppä J., Wang Y., Kukko A., Haggrén H., Yu X., Kaartinen H., Jaakkola A., Guan F. (2016). Terrestrial laser scanning in forest inventories. ISPRS J. Photogramm. Remote Sens..

[44] Olsen M.J., Kuester F., Chang B.J., Hutchinson T.C. (2010). Terrestrial laser scanning-based structural damage assessment. J. Comput. Civ. Eng..

[45] Pritchard D., Sperner J., Hoepner S., Tenschert R. Terrestrial laser scanning for heritage conservation: The Cologne Cathedral documentation project. Proceedings of the ISPRS Annals of the Photogrammetry, Remote Sensing and Spatial Information Sciences.

[46] Suchocki C. (2020). Comparison of Time-of-Flight and Phase-Shift TLS Intensity Data for the Diagnostics Measurements of Buildings. Materials.

[47] Voegtle T., Schwab I., Landes T. Influences of different materials on the measurements of a terrestrial laser scanner (TLS). Proceedings of the International Archives of the Photogrammetry, Remote Sensing and Spatial Information Sciences.

[48] Tan K., Cheng X. (2016). Correction of incidence angle and distance effects on TLS intensity data based on reference targets. Remote Sens..

[49] Alkan R.M., Karsidag G. Analysis of The Accuracy of Terrestrial Laser Scanning Measurements. Proceedings of the FIG Working Week 2012-Knowing to Manage the Territory, Protect the Environment, Evaluate the Cultural Heritage.

[50] Lichti D., Stewart M., Tsakiri M., Snow A.J. (2000). Benchmark Tests on a Three-Dimensional Laser Scanning System. Geomat. Res. Australas..

[51] Lichti D.D. (2010). A review of geometric models and self-calibration methods for terrestrial laser scanners. Bol. Ciencias Geod..

[52] Li X., Li Y., Xie X., Xu L. (2018). Lab-built terrestrial laser scanner self-calibration using mounting angle error correction. Opt. Express.

[53] Medić T., Kuhlmann H., Holst C. Automatic In-Situ self-calibration of a panoramic TLS from a single station using 2d keypoints. Proceedings of the ISPRS Annals of the Photogrammetry, Remote Sensing and Spatial Information Sciences.

[54] Soudarissanane S. (2016). The Geometry of Terrestrial Laser Scanning: Identification of Errors, Modeling and Mitigation of Scanning Geometry.

[55] Lerma J.L., Garcí D. Self-calibration of terrestrial laser scanners: Selection of the best geometric additional parameters. Proceedings of the ISPRS Annals of the Photogrammetry, Remote Sensing and Spatial Information Sciences.

[56] Soudarissanane S., Lindenbergh R., Gorte B. Reducing the error in terrestrial laser scanning by optimizing the measurement set-up. Proceedings of the International Archives of the Photogrammetry, Remote Sensing and Spatial Information Sciences.

[57] Lenda G., Marmol U., Mirek G. (2015). Accuracy of laser scanners for measuring surfaces made of synthetic materials. Photogramm. Fernerkund. Geoinf..

[58] San José Alonso J.I., Martínez Rubio J., Fernández Martín J.J., García Fernández J. Comparing Time-of-Flight and Phase-Shift. the Survey of the Royal Pantheon in the Basilica of San Isidoro (León). Proceedings of the ISPRS-International Archives of the Photogrammetry, Remote Sensing and Spatial Information Sciences.

[59] Tan K., Zhang W., Shen F., Cheng X. (2018). Investigation of TLS intensity data and distance measurement errors from target specular reflections. Remote Sens..

[60] Markiewicz J., Zawieska D. (2015). Quality assessment of the TLS data in conservation of monuments. Proc. SPIE 9527 Opt. Arts Archit. Archaeol..

[61] Boehler W., Vicent M., Marbs A. Investigating Laser Scanner Accuracy. Proceedings of the XIXth CIPA Symposium.

[62] Scharf A. (2019). Terrestrial Laser Scanning for Wooden Façade-system Inspection.

[63] Kersten T., Mechelke K., Lindstaedt M., Sternberg H. Geometric accuracy investigations of the latest terrestrial laser scanning systems. Proceedings of the FIG Working Week.

[64] Valença J., Júlio E., Helder A. Application of Photogrammetry to Bridge Monitoring. Proceedings of the 12th International Conference on Structural Faults & Repair.

[65] Han J., Hong K., Kim S., Carneiro Da Silva D.D. (2012). Application of a Photogrammetric System for Monitoring Civil Engineering Structures. Special Applications of Photogrammetry.

[66] Iglhaut J., Cabo C., Puliti S., Piermattei L., O’Connor J., Rosette J. (2019). Structure from Motion Photogrammetry in Forestry: A Review. Curr. For. Rep..

[67] Piermattei L., Karel W., Wang D., Wieser M., Mokroš M., Surový P., Koreň M., Tomaštík J., Pfeifer N., Hollaus M. (2019). Terrestrial Structure from Motion Photogrammetry for Deriving Forest Inventory Data. Remote Sens..

[68] Roncella R., Forlani G., Remondino F. Photogrammetry for geological applications: Automatic retrieval of discontinuity orientation in rock slopes. Proceedings of the SPIE-The International Society for Optical Engineering.

[69] Klawitter M., Pistellato D., Webster A., Esterle J. (2017). Application of photogrammetry for mapping of solution collapse breccia pipes on the Colorado Plateau, USA. Photogramm. Rec..

[70] Gianolio S., Mermati F., Genovese G. Image-based 3D modeling for the knowledge and the representation of archaeological dig and pottery: Sant’omobono and sarno project’s strategies. Proceedings of the International Archives of the Photogrammetry, Remote Sensing and Spatial Information Sciences-ISPRS Archives.

[71] Gajski D., Solter A., Gašparovic M. Applications of macro photogrammetry in archaeology. Proceedings of the International Archives of the Photogrammetry, Remote Sensing and Spatial Information Sciences-ISPRS Archives.

[72] Waas M., Zell D. Practical 3D photogrammetry for the conservation and documentation of Cultural Heritage Introduction. Proceedings of the International Conference on Cultural Heritage and New Technologies.

[73] Remondino F., Rizzi A., Barazzetti L., Scaioni M., Fassi F., Brumana R., Pelagotti A. (2011). Review of geometric and radiometric analyses of paintings. Photogramm. Rec..

[74] Westoby M.J., Brasington J., Glasser N.F., Hambrey M.J., Reynolds J.M. (2012). “Structure-from-Motion” photogrammetry: A low-cost, effective tool for geoscience applications. Geomorphology.

[75] El-Hakim S., Beraldin J.-A., Blais F. Critical Factors and Configurations for Practical 3D Image-Based Modeling. Proceedings of the 6th Conference on Optical 3D Measurements Techniques.

[76] Nyimbili P.H., Demirel H., Şeker D.Z., Erden T. Structure from Motion (SfM)-Approaches and Applications. Proceedings of the International Scientific Conference on Applied Sciences.

[77] Bianco S., Ciocca G., Marelli D. (2018). Evaluating the Performance of Structure from Motion Pipelines. J. Imaging.

[78] Lichti D., Pfeifer N. Introduction to Terrestrial Laser Scanning. ftp://ftp.ecn.purdue.edu/bethel/tls.pdf.

[79] Vosselman G., Maas H.-G. (2010). Airborne and Terrestrial Laser Scanning. Current.

[80] Luhmann T., Robson S., Kyle S., Boehm J. (2015). Close-Range Photogrammetry and 3D Imaging.

[81] Habib A.F. Photogrammetric Bundle Adjustment. https://dprg.geomatics.ucalgary.ca/system/files/akam_531_ppt_ch1_relase-compatibility-mode.pdf.

[82] Buratowski T. Teoria Robotyki [Robotics Theory]. https://www.robotyka.com/teoria.php/teoria.71?fbclid=IwAR3wprGRF6JNb3LYXD7UsDWpQQzrcYteIIvWXEG-WVTcUjb2O8lTG2tj-To.

[83] Malissiovas G., Neitzel F., Petrovic S. (2016). Götterdämmerung over total least squares. J. Geod. Sci..

[84] Mathworks.com. http://uk.mathworks.com/help/matlab/ref/eig.html?searchHighlight=eigenvalue&s_tid=doc_srchtitle.

[85] Torr P.H.S., Zisserman A. (2000). MLESAC: A New Robust Estimator with Application to Estimating Image Geometry. Comput. Vis. Image Underst..

[86] Zhou Q.-Y., Park J., Koltun V. Open3D: A Modern Library for 3D Data Processing. http://www.open3d.org/wordpress/wp-content/paper.pdf.

